# Design, synthesis, *in-vivo*, and *in-silico* studies of 1,2,3-triazole tethered derivatives of morphine as novel anti-nociceptive agents

**DOI:** 10.1371/journal.pone.0323189

**Published:** 2025-06-16

**Authors:** Faeze Nourmandipour, Reihane Emadi, Peyman Salehi, Mona Kamelan Zargar Zarin, Mona Khoramjouy, Amirreza Dowlati Beirami, Mehrdad Faizi

**Affiliations:** 1 Department of Phytochemistry, Medicinal Plants and Drugs Research Institute, Shahid Beheshti University, Tehran, Iran; 2 Department of Pharmacology and Toxicology, School of Pharmacy, Shahid Beheshti University of Medical Sciences, Tehran, Iran; 3 Phytochemistry Research Center, Shahid Beheshti University of Medical Sciences, Tehran, Iran; 4 Department of Analytical Chemistry, Faculty of Chemistry, University of Vienna, Vienna, Austria; 5 Department of Medicinal Chemistry, School of Pharmacy, Shahid Beheshti University of Medical Sciences, Tehran, Iran; ISF College of Pharmacy Moga, INDIA

## Abstract

Due to the use of morphine as a well-known analgesic, a semi-synthesis of its newer triazole derivatives was performed in this project. Several derivatives were analyzed via molecular docking and a set of target molecules with acceptable docking scores were selected for the synthesis. The project focused on targeting one of the pharmacophores of morphine. The phenolic hydroxy group of morphine was reacted with propargyl bromide to furnish the terminal alkyne. This compound, as starting material for the click reaction underwent 1,3-dipolar cycloaddition reaction with different azides to produce the target 1,2,3-triazole tethered derivatives of morphine. The anti-nociceptive properties of the products were evaluated by tail flick test. It was observed that compounds **3b**, **3d**, and **3k** with (ED_50 _= 0.23 mg/kg) showed superior pain relief activities in comparison with morphine (ED_50_ = 0.69 mg/kg). Finally, computational ADME/T studies were performed via SwissADME web server to gain a better understanding of the pharmacokinetics of the synthesized compounds in humans.

## Introduction

Pain is an unpleasant sensation and an emotional experience mostly associated with real tissue damage. Pain motivates humans to keep away from potentially destructive conditions, protect the damaged part of the body until recovery, and prevents the conditions in the future [1]. In the pathophysiology of pain, there is a very complex relationship between peripheral and central structures from the surface of skin to the cortex of the brain. One of the most effective classes of drugs for pain relief are opioid pain relievers [2].

Opioids, derived from the poppy plant, are natural narcotics and alkaloids that bind to opioid receptors, producing effects similar to endorphins. These pain-killing neurotransmitters, like endorphins, reduce pain in the body by affecting the central nervous system [3]. However, chronic use of these drugs leads to a decrease in analgesic responses, tolerance and dependence. The tolerance of the body to opioid substances is a pharmacodynamic process that includes neural adaptive mechanisms. Many messenger pathways are intracellular. Opioids themselves harm the body’s organic system. They do not deliver much, because they only affect their specific receptors, however, constant and long-term use of these substances can be accompanied by physical damage that causes undesirable effects. Unwanted effects of opioids include addiction, nausea, dizziness, drowsiness, constipation, low blood pressure, sweating, itching, etc. [3,4]. In general, opioids include three categories of natural narcoticizes, semi-synthetic alkaloids derived from the poppy plant, synthetic substitutes with similar pharmacological properties, and endogenous peptides [5]. Opioid receptors are proteins with a neural locus that opioid compounds bind to produce a response. These receptors include four general categories including μ, δ, κ, and opioid receptor-like 1 (ORL-1) [6]. Most of the existing pain relievers act on the μ receptor. As a result of morphine consumption, analgesia as well as euphoria, weakening of breathing and addiction are mainly caused by the effect on μ receptors. Although δ and κ receptors can contribute to analgesia, it is not known how much morphine binding to these two receptors contributes to its analgesic effect [7].

**Fig 1:**
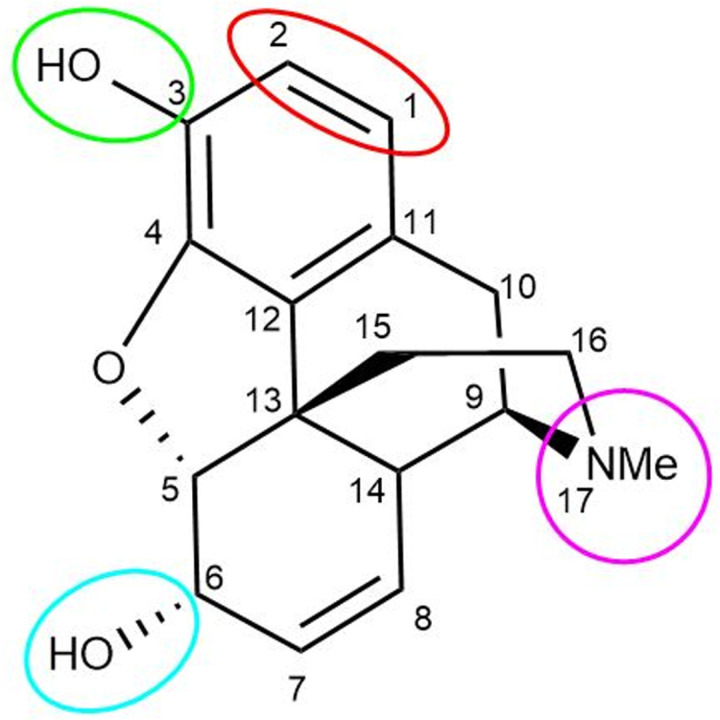
Sites of modification on the morphine molecule.

Morphine is the first identified substance in the large group of morphinans, which was purified from the raw source of opium by Freidrich Wilhelm Adam Serturner about two hundred years ago. This compound is the most abundant opioid derived from opium and is still known as the standard substance for comparing the analgesic activities of the other opioids [5,8]. Morphine is a complete agonist for the μ opioid receptor and creates its analgesic effect by binding to this receptor in the central and peripheral nervous system [9]. Morphine is a benzyl isoquinoline alkaloid [10] and the studies conducted on the structure of morphine, have determined that this compound has several important active regions, which can be manipulated to synthesize different derivatives by placing different functional groups on them [11] (Fig 1).

Morphine derivatives with modifications at positions 1, 4, 6 [12–14], and 3 [15,16] showed analgesic properties and high binding affinity to μ, κ, and δ receptors.

The 1,3-dipolar cycloaddition between azides and alkynes was first performed by Michael in 1893 and further studies by Huisgen in 1960–1980 were reported. The enormous gap in this research between 1893 and 1960 can be attributed to several factors, including limited chemical tools and techniques, competing research priorities, lack of selectivity, and technological constraints [17]. The products of the Huisgen reaction are 1,4- and 1,5-triazoles, which are carried out under different thermal and catalytic conditions [18]. A triazole compound has numerous pharmacological activities, including anticancer effects associated with its thiazolidine-4-one scaffold, anti-inflammatory and anti-nociceptive properties, as well as antibacterial, antifungal, antibiotic, antiviral, anti-diabetic, and anti-protozoal activities [19–21]. In 2002, the research groups of Fokin and Sharpless [22] and Meldahl et al [23], separately introduced copper (I)-catalyzed impressive azide-alkyne cycloaddition [24]. Under the guise of Click Chemistry Carolyn R. Bertozzi, K. Barry Sharpless, and Morten Meldal received the Nobel Prize in Chemistry in 2022 for simplifying of complex processes [25]. The potential application of this reaction is too high, as the azide and alkyne components can be combined in a wide variety of different substituents [26]. The azide-alkyne cycloaddition reaction catalyzed by Cu(I) (CuAAC) is a click reaction that leads to the formation of the triazole ring [27]. Cu(II) salt can be used to facilitate the reaction of the pre-catalyst and usually CuSO_4_ is used with a reducing agent such as sodium ascorbate [28,29]. The 1,2,3-triazole ring is an active group in peptide bond simulation and drug discovery. This part is an amide bond bioisoster with great similarity in its structure and electronic characteristics, which has great stability against metabolic changes [30]. Another feature of these rings is the resistance to hydrolysis in biological environments and the active component in binding to the biological target [31]. 1,2,3-Triazole rings have many biological activities of their own, which is due to the ability of dipolar interactions, as well as the creation of hydrogen bonds. Among these properties are anti-cancer [32], anti-inflammatory, anti-tuberculosis, anti-malarial, anti-fungal, anti-microbial, and anti-HIV [22,23,33].

In this study, we designed and synthesized 14 morphine derivatives targeting its phenolic-OH group. We investigated the interactions of these compounds with μ opioid receptor by molecular modeling techniques and then synthesize triazole-tethered derivatives of morphine by CuAAC. Ultimately, we evaluated the anti-nociceptive effects of the compounds through *in vivo* tests in mice, and performed a computational ADME/T test to estimate the disposition of the compounds in the body.

## Results and discussion

### Molecular modeling

**Fig 2:**
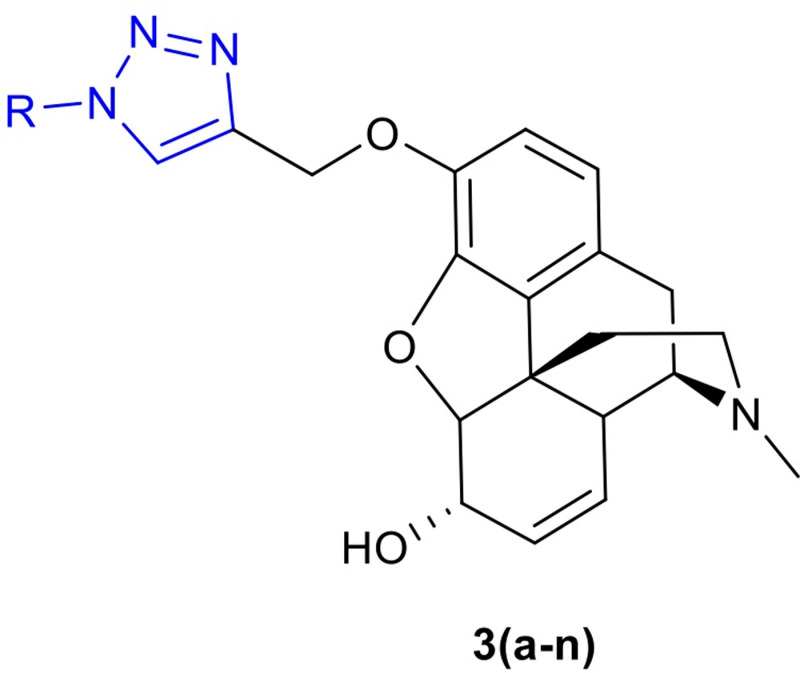
The structure of the designed 1,2,3-triazole-tethered derivatives of morphine.

Computer-assisted drug design has been a helpful and cost-effective strategy to utilize the drug discovery process [34]. In this study, a structure-based drug design approach was implemented by molecularly docking potential μ receptor agonists against the crystal structure of active-μ receptor optioned from PDB (PDB code: 5C1M). The core structure of the targeted derivatives is shown in Fig 2. Our goal in creating these compounds was to enhance the effect of triazole ring by using both electron-donating and electron-withdrawing groups. Molecular docking was performed to investigate the affinity of the designed derivatives towards the active site of μ receptor before synthesis. Studies have shown that the binding site of morphine has the same cavity that Bu72 agonist morphine ligands interact with, and thus, we selected this cavity as the reference for docking studies [35,36]. As the result of the molecular docking study, Table 1 shows the docking scores for the best pose of each compound with the highest docking score. After docking, the analysis of key interactions was performed via Discovery Studio visualizer 21.1.0.20298.

**Table 1:** The result of docking studies of compounds along by their docking score using AutoDock Vina and DOCK 6.11.

Entry	Compound No.	R	Dock score (Vina)	Dock Score (DOCK)
1	**3a**	4-ClC_6_H_4_-	-7.7	-27.32
2	**3b**	3,4-Cl_2_C_6_H_3_-	-9.1	-25.46
3	**3c**	3-EtC_6_H_4_-	-7.4	-28.61
4	**3d**	4-FC_6_H_4_-	-9.2	-43.63
5	**3e**	3,5-Me_2_C_6_H_3_-	-8.1	-43.00
6	**3f**	4-EtC_6_H_4_-	-7.7	-31.09
7	**3g**	2-EtC_6_H_4_-	-8.3	-30.82
8	**3h**	4-MeOC_6_H_4_-	-7.5	-32.45
9	**3i**	4-MeC_6_H_4_-	-7.9	-36.75
10	**3j**	C_6_H_5_-	-7.5	-38.68
11	**3k**	C_6_H_5_CH_2_-	-9.7	-37.29
12	**3l**	4-FC_6_H_4_CH_2_-	-7.4	-45.10
13	**3m**	4-BrC_6_H_4_CH_2_-	-8.0	-38.92
14	**3n**	4-MeC_6_H_4_CH_2_-	-7.0	-40.74
15	**Morphine**	–	-8.4	-41.28

**Fig 3:**
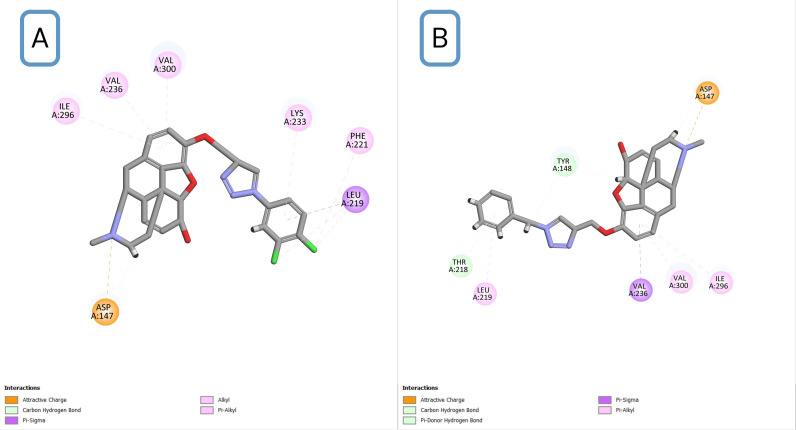
2D interaction diagrams of two designed ligands. (A) and (B) are 3b and 3k derivatives, respectively.

By considering N-Asp147 interaction as the key pharmacophore, other interactions were mainly at the “a site” of the active site [37]. It is worth noticing that a study by Kaserer et al. suggests that the interactions with Asp147 and Tyr148 are essential for ligands to bind to the receptor [38]. In this case, in most of the designed compounds, the triazole ring interacted with Tyr148 with a π donor hydrogen bond. In addition, there were some new recurrent interactions between the receptor and the tethered fragment. Table 2 shows a summary of common interactions and Fig 3 shows an example of 2D interactions.

**Table 2:** Common interactions of the designed compounds resulted from docking studies.

Binding site	Interacting amino acids
n site	ASP147
a site	Tyr148, Ile296, Val236, and Val300
Main hydrophobic interactions of aryl azide fragment of compounds	leu232, lys233, leu219, his54, and phe221

Based on docking scores, analysis of docking poses, and consideration of reported key interactions, 14 novel morphine derivatives were introduced as potential candidates for further investigation. Docking results from this study showed that all the novel compounds can form polar interactions with Asp147 residue. Furthermore, interactions with Tyr148 and His297 were observed in the most of the candidates. It is worth noting that a slight configuration change was observed, which could be due to the small capacity of the phenolic binding site [38]. The typical docking poses revealed that the ligands can interact with the receptor through tertiary nitrogen. The phenolic end of the aryl azide fragment points towards the lipophilic pocket, also known as the a-site, which includes residues like Lys233, His54, and Val300. Finally, the triazole ring of the aryl azide fragment and the aliphatic hydroxyl group of the core, approach Tyr148 and form hydrophilic bonds in most compounds. Altogether, the pattern of docking pose of compounds in this study is slightly different than the conventional pattern, but it forms all necessary interactions. In this project, the phenolic hydroxy group of morphine was selected for chemical reaction. According to the synthetic route designed in this project, it was reacted with propargyl bromide, and the terminal alkyne was obtained. Further, by reacting propargylated morphine with different azides, various triazole compounds were synthesized. The synthesis of triazole compounds was done through the click reaction and then the molecular simulation of the synthesized derivatives with its target receptor was carried out. Among all the synthesized derivatives, compounds **3b**, **3d**, and **3k** showed an excellent affinity to bind to the active site of μ receptor.

### Ligand-receptor complex molecular dynamics simulation

#### Docking validation.

Validation of the docking protocol was conducted via redocking the co-crystal ligand. A comparison of the experimental and docked poses yielded a maximum alignment with RMSD = 0.37, confirming the accuracy of the docking setup. Following this, ligand structures were converted to mol2 format using OpenBabel, and docking simulations were performed as previously described [39]. Additionally, post-docking, the interactions of the compounds with the receptor were analyzed using Ligplot, and Both structures formed key interactions [40].

#### MD simulation validation.

The system’s temperature throughout the simulation remained stable with minor fluctuations, indicating that the system achieved its equilibrium temperature. Also, pressure, a fluctuating parameter during MD simulations, showed substantial variability during the equilibration phase. The potential energy graphs exhibited a plateau with minimal fluctuations, indicating stability. For the morphine complex, the mean potential energy was -956,193.0682 kJ/mol, while for the 3d complex, it was -966,132.7145 kJ/mol. Both systems maintained consistent potential energy throughout the simulation. Also, The total energy analysis showed convergence for the systems at -671,583.7205 kJ/mol and -679,052.6067 kJ/mol, respectively. Convergence of total energy confirmed that the systems reached equilibrium before further analysis. Consistent with this, the system volumes converged at approximately -1,019.639932 nm³ and -1,026.385828 nm³, indicating structural equilibrium.

#### MD simulation results.

**Fig 4:**
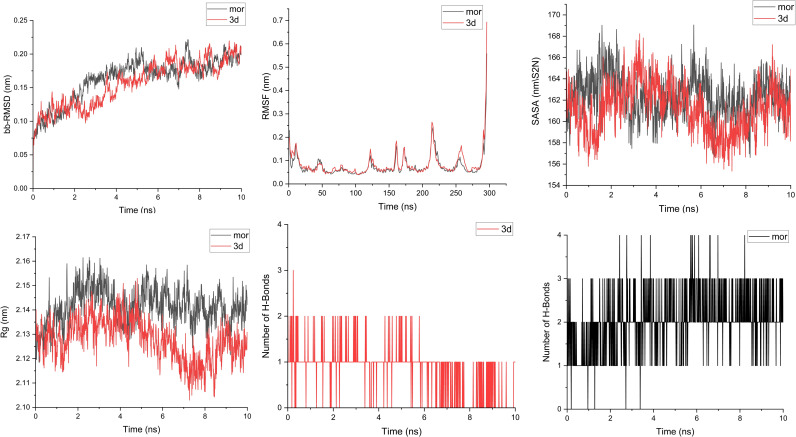
Molecular dynamic simulation of compound 3d and Morphine against active- μ receptor optioned from Protein Data Bank (PDB code: 5C1M).

**Fig 5:**
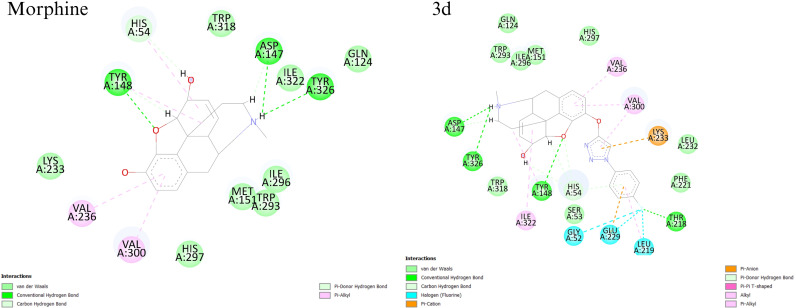
interactions of the of active mu-opioid receptor in complex with Morphine and 3d after molecular dynamic simulation for 10ns.

The root means square deviation (RMSD) analysis was conducted to evaluate the structural stability of the protein-ligand complexes. RMSD calculations were performed for the protein backbone, and both systems showed gradual RMSD increases, stabilizing after approximately six ns with minimal fluctuations, indicating relative equilibrium. RMSD profiles were similar for both systems. Root mean square fluctuation (RMSF) was calculated to assess the flexibility of protein residues. Higher RMSF values indicated greater flexibility. Both systems demonstrated similar patterns, though the morphine complex exhibited slightly lower RMSF values, suggesting greater stability. The radius of gyration was analyzed to understand structural compactness. For the morphine complex, Rg initially increased, stabilized at 3 ns, and fluctuated minimally until the end, indicating a stable fold, and for the 3d complex, Rg decreased between 4–7 ns, reflecting more compaction compared to the morphine. However, it showed more fluctuations. Solvent Accessible Surface Area (SASA) values, indicating solvent exposure, fluctuated between 155–169 nm² for both systems and converged toward the initial values. These results showed no significant structural opening or closing throughout the simulation. Finally, hydrogen bonds were evaluated, as they play critical roles in protein-ligand interactions and specificity. Morphine formed a stable network of 1–4 hydrogen bonds, with 3 persisting throughout the simulation, and the 3d ligand also exhibited 1–3 hydrogen bonds, with one bond persisting until the end. Fig 4 shows the results of the simulation. Also, non-hydrogen bond interactions were analyzed using Discovery Studio. The final simulation frame revealed more interactions between the receptor and the 3d ligand due to its extended structure and numerous aromatic rings Fig 5.

### Chemical synthesis

**Fig 6:**
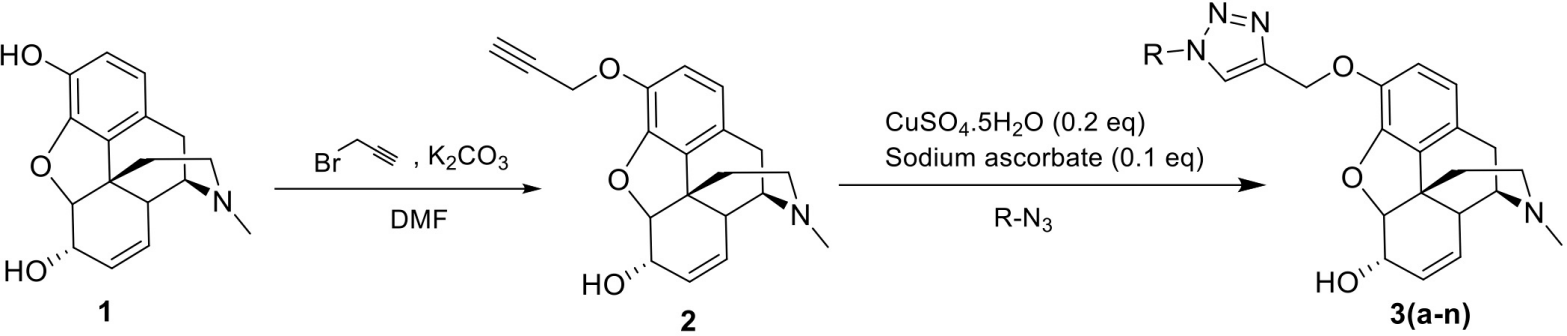
Synthesis of 1,2,3-triazole tethered derivatives of morphine.

Our strategy for the synthesis of the target molecules is shown in Fig 6.

**Fig 7:**
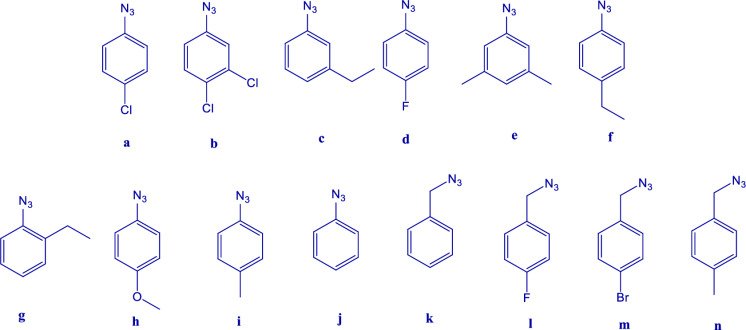
Structures of the azides (RN_3_) used for the click reaction.

According to the synthetic route designed in this project, the phenolic hydroxy group of morphine was reacted with propargyl bromide and the needed terminal alkyne for click reaction was obtained (**2**). Further, various triazole compounds were synthesized by 1,3-dipolar cycloaddition in the presence of CuSO_4_ and sodium ascorbate (**3a-n**). Triazole derivatives with a wide and diverse range of azides were synthesized to investigate the effect of different substituents on the biological properties of the products (Fig 7).

Aryl azides such as phenyl azide or its derivatives with electron-donating groups such as methoxy, methyl, ethyl, and also electron-withdrawing substituents including different halides were used. In addition, benzyl azide and its derivatives were used as another group of starting materials.

### Anti-nociceptive activity

**Fig 8:**
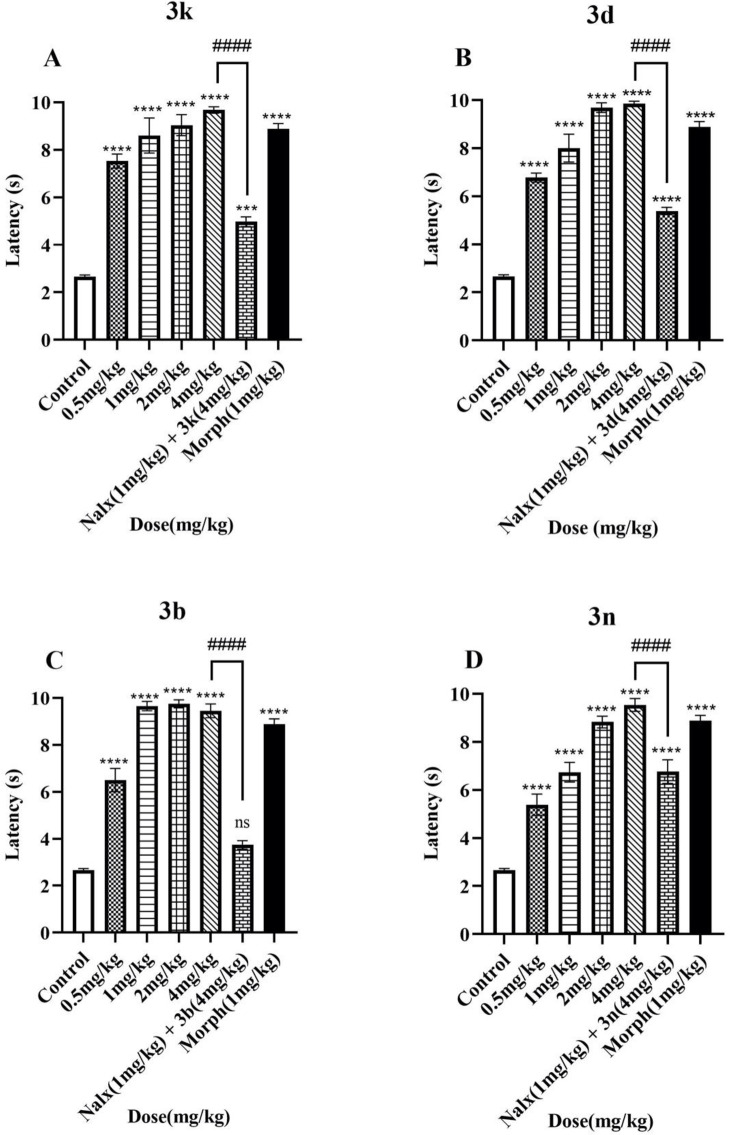
Comparison of the anti-nociceptive effects of four triazole derivatives of morphine with negative and positive control groups in the tail-flick test is demonstrated in (A) 3k, (B) 3d, (C) 3b, and (D) 3n. Results are indicated as Mean± SEM of 8 separate animals in each group. ****p ≤ 0.0001, ####p ≤ 0.0001.

Morphine is the main alkaloid in opium and acts on the central nervous system and shows analgesic properties. The main objective of this study was to design novel morphine derivatives and evaluate their anti-nociceptive activity through the tail-flick test, one of the most famous *in-vivo* experiments for acute pain assessment [41,42]. All of the compounds in all measured doses (0.5–4 mg/kg) increased the tail flick latency significantly and showed anti-nociceptive effects. Also, ED_50_ values of the novel compounds were calculated between 0.23 and 0.60 mg/kg which were lesser than ED_50_ of morphine (0.69 mg/kg) and showed higher efficacies than the positive control group. Compounds **3b, 3d,** and **3k** were the most potent with ED_50_ values of 0.23 (0.15 to 0.32), 0.23 (0.19 to 0.28), and 0.23 (0.14 to 0.34) mg/kg whereas compound **3n** showed the least efficacy with ED_50_ value of 0.60 (0.46 to 0.75) mg/kg among the fourteen novel triazole derivatives. The calculated ED_50_ values are represented in Table 3. The results of the tail-flick test for different concentrations of the powerful and the weak compounds among 14 semi-synthesized derivatives are shown in Fig 8.

**Table 3:** ED_50_ of compounds with 95% confidence interval.

Entry Molecule	ED_₅₀_ mg/kg (95% CI) *
1 **3a**	0.45 (0.33 to 0.59)
2 **3b**	0.23 (0.17 to 0.31)
3 **3c**	0.51 (0.37 to 0.70)
4 **3d**	0.23 (0.19 to 0.28)
5 **3e**	0.29 (0.19 to 0.37)
6 **3f**	0.25 (0.19 to 0.31)
7 **3g**	0.31 (0.22 to 0.40)
8 **3h**	0.50 (0.38 to 0.63)
9 **3i**	0.36 (0.28 to 0.45)
10 **3j**	0.43 (0.33 to 0.55)
11 **3k**	0.23 (0.14 to 0.34)
12 **3l**	0.28 (0.20 to 0.37)
13 **3m**	0.24 (0.12 to 0.39)
14 **3n**	0.60 (0.46 to 0.75)
15 **Morphine**	0.69 (0.58 to 0.83)

* CI= Confidence Interval.

It is important to note that naloxone significantly reduced the anti-nociceptive effects of the compounds in the tail-flick test. (10 additional graphs of other compounds can be found in the supporting information). This leads to the conclusion that the synthesized compounds exhibited their anti-nociceptive activity through µ opioid receptors.

Considering all aspects, the results of this test indicated that all of the compounds had anti-nociceptive effects and showed better efficacy than the parent compound of morphine. Their interaction with the μ receptor seems responsible for this result due to the tail-flick latency reduction due to naloxone pre-consumption. In this case, docking results also align with the biological evaluations. Compounds **3b**, **3d**, and **3k** not only showed the highest efficacies but also they exhibited the highest docking scores.

### ADME/T studies for triazole derivatives

The results of ADME/T study using the SwissADME web server showed that all of the novel compounds could benefit from acceptable ADME/T properties and indicate minimal violations of drug-likeness criteria and no alerting moieties. Physicochemical properties of the structures, such as molecular weight, log P, H-bond donors, and H-bond acceptors, were evaluated and are presented in Table 4. Based on SwissADME predictions, morphine can cross the blood brain barrier (BBB) without a transporter and it has the advantage of getting transferred to the central nervous system by being a P-glycoprotein substrate [43]. Nevertheless, the synthesized triazole-derivatives of morphine have better BBB and GI penetration compared to morphine without needing any transporter. In this case, based on the biopharmaceutical classification system (BCS) they can be categorized as BCS Class II drugs. This can make them ideal candidates for lipid nanoparticle (LNP) formulations [44]. Based on the ADME/T findings, lipophilic fragments in the majority of the designed compounds enhance BBB penetration independently. Moreover, these compounds can potentially inhibit CYP2C9, CYP2D6, and CYP3A4.

**Table 4:** Physicochemical properties of synthesized compounds calculated by the SwissADME web server.

Molecule	MW	Rotatable bonds	H-bond acceptors	H-bond donors	TPSA	XLOGP3
3a	476.95	4	6	1	72.64	3.75
3b	511.4	4	6	1	72.64	4.37
3c	470.56	5	6	1	72.64	3.91
3d	460.5	4	7	1	72.64	3.22
3e	470.56	4	6	1	72.64	3.85
3f	470.56	5	6	1	72.64	3.91
3g	470.56	5	6	1	72.64	3.91
3h	472.54	5	7	1	81.87	3.09
3i	456.54	4	6	1	72.64	3.48
3j	442.51	4	6	1	72.64	3.12
3k	456.54	5	6	1	72.64	3.05
3l	474.53	5	7	1	72.64	3.15
3m	535.43	5	6	1	72.64	3.74
3n	470.56	5	6	1	72.64	3.42
Morphine	286.35	0	3	2	54.13	0.76

## Conclusion

Although morphine is one of the most important pain relievers in the world, an enormous effort has been devoted for the synthesis of new analogues with a higher efficacy and lower side effects [11]. Potential drug-drug interactions are a primary concern when considering CYP enzyme inhibition by morphine derivatives. Morphine is primarily metabolized by UDP-glucuronosyltransferase (UGT) enzymes, with limited involvement of CYP enzymes. However, modifications like triazole binding may alter the metabolic profile, that necessitates careful monitoring or dose adjustment in patients taking other medications. Hybridization of triazole ring with many drugs and lead compounds have shown to be a reasonable strategy to achieve these goals [45–46]. In this context, we designed 14 triazole derivatives of morphine, which interact with the active site of μ opioid receptor and have affinity comparable to that of morphine. Synthesis of the target molecules by a 1,3-dipolar cycloaddition click reaction ended up with the formation of a diverse library of morphine tethered triazole derivatives. The *in vivo* experiment confirmed that the novel compounds have efficacy and show anti-nociceptive effects and most of them are even more potent than morphine. These observations show that by placing polar heterocycles in the position 3 of morphine, better results could be obtained that may open a new horizon for the future researches.

## Materials and methods

### Molecular docking

The crystallographic structure of 5C1M (active μ opioid receptor bound to BU72) was obtained from the Protein Data Bank and prepared for docking by applying charge, adding polar hydrogen, and removing ligands and water via AutoDock Vina 1.1.2 software [47]. The designed compounds were also prepared via AutoDock Vina 1.1.2 software. Afterward, the docking calculations were performed in a grid box of 14 × 14 × 14 Å, centered at x = 1.285656, y = 16.447875, and z = -59.114250. Exhaustiveness and the number of modes were set to 100 and 20, respectively. Considering the interaction of the basic nitrogen (N) with Asp147 as the most crucial interaction, proper positions were selected for the investigation of interactions via Discovery Studio visualizer 21.1.0.20298. As a complementary and conformative approach, the DOCK 6.11 software was also employed, which uses a systematic search algorithm and knowledge-based scoring functions [48]. For this, a semi-flexible docking approach, where the ligands were flexible, and the receptor remained rigid, performed with similar settings for the grid box.

### Ligand-Receptor Complex Molecular Dynamics Simulation

Docking consideres the receptor to be rigid, which may affect the precision of the result. To simulate realistic conditions, Molecular Dynamics (MD) simulations were conducted Using GROMACS 2024.4 to evaluate system dynamics, stability, and predicted interactions over time [49].

To investigate the dynamic interaction of synthesized compounds compared to Morphine, molecular dynamic (MD) simulations were performed using 3d, as a promising compound from the docking studies, and Morphine, as a well-known agonist. For docking simulations, the DOCK 6.11 software was employed as previously described [48]. For the protein, the structure was obtained from the Protein Data Bank (PDB code: 5C1M). Chimera software was used to clean the protein structure by removing chain B, water molecules, and all ligands in preparation. Hydrogen atoms and atomic charges were added, and the resulting structure was saved in mol2 format. A duplicate of the structure was created without hydrogen atoms for molecular surface generation. Using the sphere generation algorithm, spheres were created on the protein surface to represent binding pockets, which were clustered. Ligand binding site spheres within a 10 Å radius of the crystallized ligand were selected for search space determination.

The CHARMM-GUI web server was used to construct the simulation system. Protein-ligand complexes were uploaded via the Membrane Builder module, and PPM2 was employed to determine receptor orientation in the membrane [50]. The system included a Lipid bilayer of 128 POPC molecules in each layer, Water, and ions for neutralizing the system. For the forcefield, CHARMM36 was applied for topology and parameter files.

Afterwards, the system underwent energy minimization using steepest descent (SD) method to eliminate spatial clashes and achieve stability. Minimization continued until the maximum force was less than 1000 kJ/mol/nm. Equilibration was performed in two steps of NVT ensemble, stabilized system at a temperature of 310 K, and NPT ensemble, Stabilizing system pressure and density using the Parrinello-Rahman barostat. Following equilibration, MD simulations were executed for 100 ns using the GROMACS 2024 software with the full-atom CHARMM force field.

### Chemistry

Chemicals and solvents were purchased from Merck, Fluka, and Sigma-Aldrich which were used without any further purification. Pharmaceutical-grade morphine was purchased from Faran Chemi Pharmaceutical Company. Progress of reactions was monitored on silica gel 60 F254 (Merck) plates and blots were observed under UV light. Silica gel 60 (particle size 0.200–0.063 μm, 230–70 mesh) was used for the column chromatography. ^1^HNMR and ^13^C NMR spectra were obtained on a Bruker and were taken at the frequencies of 125 MHz for carbon and 500 MHz for hydrogen. NMR spectra were run in CDCl_3_ as a solvent. Signal multiplicities are reported as: s = singlet; d = doublet; t = triplet; dd = doublets of doublet; m = multiplet. Coupling constants (*J*) are reported in Hertz (Hz). HRESI-MS spectra in positive ion mode was recorded on a Bruker micro-TOFESI-MS system with a scan range of m/z 200–1500. Azides were synthesized according to literature methods [51]. HPLC analysis was performed on a waters system (United States, Massachusetts) equipped with a binary pump (waters2695 alliance separation modul with one pairs of pump heads of 10). The systetem also featured a PDA detector (waters2696). A hilic bare silica which was purchased from Kherad.Azma.Co (Iran, Tehran) with a particle size of 5μm as a stationary phase was packed in stainless steel column (4.6 mm I.D. × 250 mm I.D.).

### Propargylation of morphine

*o-Propargyl morphine, (4R,4aR,7S,7aR,12bS)-3-methyl-9-(prop-2-yn-1-yloxy)-2,3,4,4a,7,7a-hexahydro-1H-4,12-methanobenzofuro[3,2-e]isoquinolin-7-ol*

Morphine (**2**; 1mmol, 4g) was dissolved in DMF (5 mL) and stirred for 15 minutes at room temperature until it became a clear solution. Potassium carbonate (2 mmol, 3.60 g) was slowly added to the mixture. After 10 minutes, propargyl bromide (1.2 mmol, 960 μL) was added dropwise. The reaction was stirred for 24 hours at room temperature. After the completion of the reaction, the DMF solvent was removed by a rotary evaporator under reduced pressure and the remaining material was extracted by ethyl acetate (3 × 150 mL). The organic phase was dried with sodium sulfate and then concentrated under reduced pressure. Finally, the synthesized compound was purified by flash chromatography on silica gel with hexane-ethyl acetate as eluent. The product was obtained in 70% yield.

1 H NMR (500 MHz, CDCl_3_) (δ, ppm):6.79 (d, *J* = 8.0 Hz, 1H, H_Ar_), 6.61 (d, *J* = 8.0 Hz, 1H, H_Ar_), 6.06 (dd, *J* = 9.5, 9.2 Hz, 1H, H_Olefin_), 5.83 (d, *J* = 9.5 Hz, 1H, H_Olefin_), 5.22 (d, *J* = 6.3 Hz, 1H, O-CH-CH-OH), 4.82 (d, *J* = 2.2 Hz, 2H, O-CH_2_), 4.30–4.25 (m, 1H, CH-OH), 3.76 (s, 1H, H_Alkyne_), 2.91–2.88 (m, 1H, C_Ar_-CH_2_), 2.81 (s, 1H, C_Ar_-CH_2_), 2.74 (s, 2H, N-CH_2_), 2.56 (s, 1H, N-CH-), 2.43 (s, 3H, N-CH_3_), 2.33 (s, 1H, C_q_-CH-CH-), 2.08 (s, 1H, N-CH_2_-CH_2_-), 1.98 (m, 1H, N-CH_2_-CH_2_-).^13^C NMR(125 MHz, CDCl_3_) (δ, ppm): 141.23, 132.10, 129.46, 127.53, 125.19, 122.77, 117.87, 90.41, 75.72, 73.35, 66.17, 63.72, 57.45, 51.40, 45.49, 45.35, 41.98, 37.19, 33.14. HRMS (ESI): [M + H]+ C_20_H_21_NO_3_ calcd. 324.1598, found 324.0690.

### General procedure for the synthesis of triazole derivatives

**2** (1mmol) was dissolved in methanol (3mL). Then sodium ascorbate (0.4mmol, 0.12g) was added to the reaction along with copper sulfate (0.2mmol, 0.15g). After 10 minutes, 1 mmol of the azide was added to the flask and the reaction was stirred at room temperature by a magnetic stirrer. After 15–45 minutes, the solvent was evaporated under vacuum using a rotary evaporator. On the remaining, ammonia solution (5 mL) was added and the product was extracted with dichloromethane (3 × 100 mL). The resulting compounds were purified by column chromatography on silica gel.

*(4R,4aR,7S,7aR,12bS)-9-((1-(4-chlorophenyl)-1H-1,2,3-triazol-4-yl)methoxy)-3-methyl-2,3,4,4a,7,7a-hexahydro-1H-4,12-methanobenzofuro[3,2-e]isoquinolin-7-ol (Compound* ***3a***)

Yield:64%, oil. ^1^H NMR (500 MHz, CDCl_3_) (δ, ppm):7.92 (s, 1H,H_Triazole_), 7.51 (d, *J* = 2.3 Hz, 2H,H_Ar_), 7.49 (d, *J* = 2.2 Hz, 2H,H_Ar_), 6.68 (d, *J* = 8.0 Hz, 1H,H_Ar_), 6.50 (d, *J* = 8.0 Hz, 1H,H_Ar_), 6.03 (dd, *J* = 9.5, 9.3 Hz, 1H,H_Olefin_), 5.86 (d, *J* = 9.9 Hz, 1H,H_Olefin_), 5.26 (d, *J* = 6.0 Hz, 2H,O-CH_2_), 5.23 (d, *J* = 5.6 Hz, 1H, O-CH-CH-OH), 4.35–4.23 (m, 1H, CH-OH), 2.85 (m, 1H,C_Ar_-CH_2_), 2.68 (m, 1H,C_Ar_-CH_2_), 2.44–2.38 (m, 2H,N-CH_2_), 2.31 (s, 3H,N-CH_3_), 2.26–2.20 (m, 1H,N-CH-), 2.19 (s, 1H,C_q_-CH-CH-), 1.98 (d, J = 13.1 Hz, 1H,N-CH_2_-CH_2_-), 1.37–1.30 (m, 1H,N-CH_2_-CH_2_-).^13^C NMR (125 MHz, CDCl_3_) (δ, ppm): 144.10, 142.34, 138.56, 132.96, 132.72, 132.17, 131.85, 127.38, 127.32, 127.11, 124.60, 122.50, 119.12, 118.86, 88.29, 63.67, 60.03, 50.29, 49.50, 42.62, 39.53, 34.90, 30.08.HRMS (ESI): [M + H]^+^ C_26_H_25_ClN_4_O_3_ calcd. 477.1695, found 477.3673.

*(4R,4aR,7S,7aR,12bS)-9-((1-(3,4-dichlorophenyl)-1H-1,2,3-triazol-4-yl)methoxy)-3-methyl-2,3,4,4a,7,7a-hexahydro-1H-4,12-methanobenzofuro[3,2-e]isoquinolin-7-ol (Compound* ***3b***)

Yield:78%, oil. ^1^H NMR (500 MHz, CDCl_3_) (δ, ppm): 7.96 (s, 1H,H_Triazole_), 7.90–7.89 (d, *J* = 2.0Hz, 1H,H_Ar_), 7.62 (dd, *J* = 8.7,2.0 Hz, 1H,H_Ar_), 7.59 (d, *J* = 8.7Hz, 1H,H_Ar_), 6.67 (d, *J* = 8.0 Hz, 1H,H_Ar_), 6.49 (d, *J* = 8.0 Hz, 1H,H_Ar_), 6.02 (dd, *J* = 9.5, 9.3 Hz, 1H,H_Olefin_), 5.84 (d, *J* = 9.9 Hz, 1H,H_OLefin_), 5.23 (d, *J* = 5.2 Hz, 2H,O-CH_2_), 5.21 (s, 1H, O-CH-CH-OH), 4.26 (d, *J* = 2.6 Hz, 1H, CH-OH), 2.78–2.68 (m, 1H,C_A_r-CH_2_), 2.62 (s, 1H,C_Ar_-CH_2_-), 2.42–2.35 (m, 2H,N-CH_2_-), 2.31 (s, 3H,N-CH_3_), 2.28–2.21 (m, 1H,N-CH-), 2.04 (s, 1H,C_q_-CH-CH-), 1.99–1.92 (m, 1H,N-CH_2_-CH_2_-), 1.42 (s, 1H,N-CH_2_-CH_2_-).^13^C NMR (125 MHz, CDCl_3_) (δ, ppm):146.73, 145.13, 141.10, 135.78, 133.05, 132.35, 131.42, 129.69, 127.15, 125.03, 122.25, 121.39, 119.43, 117.87, 90.73, 66.16, 63.71, 62.57, 52.49, 51.60, 45.12, 41.75, 37.61, 32.27.HRMS (ESI): [M + H]^+^ C_26_H_24_C_l2_N_4_O_3_ calcd. 510.1225, found 511.2738.

*(4R,4aR,7S,7aR,12bS)-9-((1-(3-ethylphenyl)-1H-1,2,3-triazol-4-yl)methoxy)-3-methyl-2,3,4,4a,7,7a-hexahydro-1H-4,12-methanobenzofuro[3,2-e]isoquinolin-7-ol (Compound* ***3c***)

Yield: 60%, oil. ^1^H NMR (500MHz, CDCl_3_) (δ, ppm): 8.10 (s, 1H_Triazole_), 7.58 (d, *J* = 7.2 Hz, 1H,H_Ar_), 7.49 (d, *J* = 7.0 Hz, 1H,H_Ar_), 7.42 (d, *J* = 7.5 Hz, 1H,H_Ar_), 7.40 (d, *J* = 7.3 Hz, 1H,H_Ar_), 6.68 (d, J = 8.0 Hz, 1H,H_Ar_), 6.49 (d, J = 8.0 Hz, 1H,H_Ar_), 6.03 (dd, J = 9.5,9.3 Hz, 1H,H_Olefin_), 5.87 (d, *J* = 9.9 Hz, 1H,H_Olefin_), 5.25 (d, *J* = 5.9 Hz, 2H,O-CH_2_), 5.21 (d, *J* = 5.3 Hz, 1H O-CH-CH-OH,), 4.29 (s, 1H, CH-OH), 3.00–2.95 (m, 1H,C_Ar_-CH_2_), 2.93–2.88 (m, 1H,C_Ar_-CH_2_), 2.78–2.70 (m, 2H,N-CH_2_-), 2.54 (m, 2H,CH_2_), 2.34 (s, 3H,N-CH_3_), 2.18–2.13 (m, 1H,N-CH-),2.12–2.09(m, 1H,C_q_-CH-CH-),1.95–1.85(m, 1H,N-CH_2_-CH_2_-),1.62–1.51(m, 1H,N-CH_2_-CH_2_-),1.39–1.36 (t, 3H, CH_3_). ^13^C NMR (125 MHz, CDCl_3_) (δ, ppm): 146.37, 143.21, 139.21, 136.82, 132.51, 131.54, 129.61, 128.58, 127.04, 126.67, 123.17, 122.86, 120.12, 117.82, 104.15, 63.72, 62.63, 60.39, 50.42, 47.67, 46.03, 45.99, 37.46, 31.57, 22.63, 14.10. HRMS (ESI): [M + H]^+^ C_28_H_30_N_4_O_3_ calcd. 471.2390, found 471.3075.

*(4R,4aR,7S,7aR,12bS)-9-((1-(4-fluorophenyl)-1H-1,2,3-triazol-4-yl)methoxy)-3-methyl-2,3,4,4a,7,7a-hexahydro-1H-4,12-methanobenzofuro[3,2-e]isoquinolin-7-ol (Compound* ***3d***)

Yield:76%, oil. ^1^H NMR (500 MHz, CDCl_3_) (δ, ppm): 7.91 (s, 1H,H_Triazole_), 7.70 (dd, *J* = 8.2, 3.7 Hz, 2H,H_Ar_), 7.20 (dd, *J* = 8.0, 3.9 Hz, 2H,H_Ar_), 6.67 (d, *J* = 8.0 Hz, 1H,H_Ar_), 6.48 (d, *J* = 8.0 Hz, 1H,H_Ar_), 6.01 (dd, *J* = 9.5, 9.3Hz, 1H,H_Olefin_), 5.84 (d, *J* = 9.9 Hz, 1H,H_Olefin_), 5.23 (d, *J* = 6.0 Hz, 2H,O-CH_2_), 5.20 (s, 1H, O-CH-CH-OH), 4.25 (s, 1H, CH-OH), 2.83(s.1H,C_A_r-CH_2_), 2.75–2.66 (m, 1H,C_Ar_-CH_2_), 2.43–2.36 (m, 2H,N-CH_2_), 2.31 (s, 3H,N-CH_3_), 2.26–2.20 (m, 1H,N-CH-), 2.02–1.96 (m, 1H,C_q_-CH-CH-), 1.71–1.49 (m, 1H,N-CH_2_-CH_2_-), 1.43 (d, J = 11.6 Hz, 1H,N-CH_2_-CH_2_-). ^13^C NMR (125 MHz, CDCl_3_) (δ, ppm): 161.39, 146.68, 144.76, 141.15, 132.47, 130.86, 129.91, 129.65, 128.78, 127.09, 121.68, 121.46, 117.83, 116.56, 90.75, 68.15, 66.19, 62.61, 60.38, 52.49, 51.59, 45.13, 37.49, 22.63. HRMS (ESI): [M + H]^+^ C_26_H_25_FN_4_O_3_ calcd. 461.1989, found 461.4092.

*(4R,4aR,7S,7aR,12bS)-9-((1-(3,5-dimethylphenyl)-1H-1,2,3-triazol-4-yl)methoxy)-3-methyl-2,3,4,4a,7,7a-hexahydro-1H-4,12-methanobenzofuro[3,2-e]isoquinolin-7-ol (Compound* ***3e***)

Yield:69%, oil. ^1^H NMR (500 MHz,CDCl_3_) (δ, ppm):7.93 (s, 1H,H_Triazole_), 7.34 (d, *J* = 7.7 Hz, 2H,H_Ar_), 7.07 (s, 1H,H_Ar_), 6.69 (d, *J* = 8.0 Hz, 1H,H_Ar_), 6.50 (d, *J* = 8.0 Hz, 1H,H_Ar_), 6.03 (dd, *J* = 9.5, 9.2 Hz, 1H,H_Olefin_), 5.88 (d, *J* = 9.9 Hz, 1H,H_OLefin_), 5.26 (d, *J* = 6.1 Hz, 2H,O-CH_2_), 5.21 (s, 1H, O-CH-CH-OH), 4.30–4.27 (d, *J* = 2.7 Hz, 1H, CH-OH), 2.86 (s, 1H,C_Ar_-CH_2_), 2.76–2.69 (m, 1H,C_Ar_-CH_2_-), 2.50–2.45 (m, 2H,N-CH_2_-), 2.40 (s, 6H,2CH_3_), 2.38–2.33 (s, 3H,N-CH_3_), 2.29–2.24 (m, 1H,N-CH-), 2.20–2.15 (m, 1H,C_q_-CH-CH-), 2.00–1.95 (m,1H,N-CH_2_-CH_2_-), 1.28 (s, 1H,N-CH_2_-CH_2_-).^13^C NMR (125 MHz, CDCl_3_) (δ, ppm): 144.11, 141.82, 138.62, 137.14, 134.34, 134.14, 130.08, 127.94, 127.73, 127.10, 124.49, 122.50, 122.02, 119.00, 88.25, 63.67, 60.08, 50.17, 49.35, 42.57, 39.36, 34.86, 18.75, 18.73.HRMS (ESI): [M + H]^+^ C_28_H_30_N_4_O_3_ calcd. 471.2396, found 471.5386.

*(4R,4aR,7S,7aR,12bS)-9-((1-(4-ethylphenyl)-1H-1,2,3-triazol-4-yl)methoxy)-3-methyl-2,3,4,4a,7,7a-hexahydro-1H-4,12-methanobenzofuro[3,2-e]isoquinolin-7-ol (Compound* ***3f***)

Yield:73%, oil. ^1^H NMR (500 MHz, CDCl_3_) (δ, ppm):7.86 (s, 1H,H_Triazole_), 7.33 (d, *J* = 8.4 Hz, 2H,H_Ar_), 7.32 (d, *J* = 8.4 Hz, 2H,H_Ar_), 6.68 (d, *J* = 7.9 Hz, 1H,H_Ar_), 6.48 (d, *J* = 7.9 Hz, 1H,H_Ar_), 6.03–5.98 (dd, *J* = 9.5,9.4,1H,H_Olefin_),5.86 (d, *J* = 9.9 Hz, 1H,H_Olefin_), 5.24 (d, *J* = 5.3 Hz,2H,O-CH_2_), 5.21 (s, 1H, O-CH-CH-OH), 4.25 (s, 1H, CH-OH), 2.84 (s, 1H,C_A_r-CH_2_), 2.81–2.74 (m, 1H,C_Ar_-CH_2_), 2.74–2.67 (m, 2H,N-CH_2_-), 2.40–2.35 (m, 2H,CH_2_), 2.29 (s, 3H,N-CH_3_), 2.25–2.21 (m, 1H,N-CH-), 2.21(m, 1H,C_q_-CH-CH-), 2.05 (s, 1H,N-CH_2_-CH_2_-), 2.00–1.93 (m, 1H,N-CH_2_-CH_2_-),1.29–1.27(m, 3H,CH_3_). ^13^C NMR (125 MHz, CDCl_3_) (δ, ppm): 145.04, 142.73, 136.24, 132.57, 132.19, 130.10, 129.68, 129.06, 127.10, 125.07, 124.61, 121.46, 120.57, 117.78, 90.83, 70.52, 66.59, 66.25, 63.71, 62.71, 45.17, 42.07, 37.38, 32.70, 28.44, 15.37. HRMS (ESI): [M + H]^+^ C_28_H_30_N_4_O_3_ calcd. 470.2396, found 471.5822.

*(4R,4aR,7S,7aR,12bS)-9-((1-(2-ethylphenyl)-1H-1,2,3-triazol-4-yl)methoxy)-3-methyl-2,3,4,4a,7,7a-hexahydro-1H-4,12-methanobenzofuro[3,2-e]isoquinolin-7-ol (Compound* ***3g***)

Yield:60%, oil. ^1^H NMR (500MHz, CDCl_3_) (δ, ppm):7.76 (s, 1H,H_Triazole_), 7.37 (d, *J* = 7.7 Hz, 1H,H_Ar_), 7.34 (d, *J* = 7.6 Hz, 1H,H_Ar_), 7.30 (d, *J* = 7.5 Hz, 1H,H_Ar_), 7.25 (d, *J* = 7.7 Hz, 1H,H_Ar_), 6.64 (d, *J* = 8.0 Hz, 1H,H_Ar_),6.45 (d, *J* = 8.0 Hz, 1H,H_Ar_), 6.01 (dd, *J* = 9.5, 9.3 Hz, 1H,H_Olefin_), 5.85 (d, *J* = 9.9 Hz, 1H,H_Olefin_), 5.22 (d, *J* = 5.5 Hz, 2H,O-CH_2_), 5.14 (d, *J* = 5.3Hz, 1H, O-CH-CH-OH), 4.24 (s, 1H, CH-OH), 2.82–2.80 (s,1H,C_Ar_-CH_2_), 2.78–2.72 (m,1H,C_A_r-CH_2_), 2.49 (m, 2H,N-CH_2_-), 2.42 (m, 2H,CH_2_), 2.36 (s, 3H,N-CH_3_), 2.33–2.31 (m, 1H,N-CH-), 2.30–2.29(m,1H,C_q_-CH-CH-), 2.25 (s, 1H,N-CH_2_-CH_2_-), 2.03–1.97 (m, 1H,N-CH_2_-CH_2_-), 1.12–1.07 (m, 3H,CH_3_).^13^C NMR (125 MHz, CDCl_3_) (δ, ppm):146.99, 143.69, 140.82, 139.97, 135.89, 132.74, 130.30, 130.13, 129.78, 126.74, 126.22, 125.17, 124.99, 124.84, 124.78, 117.81, 117.66, 90.85, 66.15, 62.82, 53.42, 52.62, 51.73, 44.97, 37.41, 31.22, 24.17, 14.73. HRMS (ESI): [M + H]^+^ C_28_H_30_N_4_O_3_ calcd. 470.2396, found 471.5568.

*(4R,4aR,7S,7aR,12bS)-9-((1-(4-methoxyphenyl)-1H-1,2,3-triazol-4-yl)methoxy)-3-methyl-2,3,4,4a,7,7a-hexahydro-1H-4,12-methanobenzofuro[3,2-e]isoquinolin-7-ol (Compound* ***3h***)

Yield:70%, oil. ^1^H NMR (500 MHz, CDCl_3_) (δ, ppm): 8.02 (s, 1H,H_Triazole_), 7.33 (d, *J* = 8.9 Hz, 2H,H_Ar_), 6.93 (d, *J* = 8.9 Hz, 2H,H_Ar_), 6.67 (d, *J* = 8.0 Hz, 1H,H_Ar_), 6.49 (d, *J* = 7.9 Hz, 1H,H_Ar_), 6.02 (dd, *J* = 9.4,9.2 Hz, 1H,H_Olefin_),5.86 (d, *J* = 9.9 Hz, 1H,H_Olefin_), 5.24 (d, *J* = 6.1 Hz, 1H,O-CH_2_), 5.20 (s, 1H,O-CH-CH-OH), 4.28 (s, 1H, CH-OH), 3.92 (s, 3H,OCH_3_), 2.96–2.86 (m, 1H,CAr-CH2), 2.85 (s, 1H,C_A_r-CH_2_), 2.54–2.45 (m, 2H,N-CH_2_-), 2.41 (s, 3H,N-CH_3_), 2.37–2.27 (m, 1H,N-CH-), 2.11–2.01 (m, 1H,C_q_-CH-CH-), 1.49–1.36 (m, 1H,N-CH2-CH_2_-), 1.35–1.30 (m, 1H,N-CH_2_-CH_2_-). ^13^C NMR (125 MHz, CDCl_3_) (δ, ppm):149.49, 146.70, 144.34, 141.24, 132.56, 130.46, 129.64, 126.89, 125.04, 124.54, 121.77, 117.96, 117.17, 112.45, 90.63, 66.03, 62.63, 56.26, 56.17, 52.51, 51.48, 44.97, 37.63, 31.66. HRMS (ESI): [M + H]^+^ C_27_H_28_N_4_O_4_ calcd. 472.2111, found 473.5367.

*(4R,4aR,7S,7aR,12bS)-3-methyl-9-((1-(p-tolyl)-1H-1,2,3-triazol-4-yl)methoxy)-2,3,4,4a,7,7a-hexahydro-1H-4,12-methanobenzofuro[3,2-e]isoquinolin-7-ol (Compound* ***3i***)

Yield:60%, oil. ^1^H NMR (500 MHz, CDCl_3_) (δ, ppm): 7.99 (s, 1H.H_Triazole_), 7.61 (d, *J* = 8.0 Hz, 2H,H_Ar_), 7.58 (d, *J* = 8.2 Hz, 2H,H_Ar_), 6.68 (d, *J* = 8.0 Hz, 1H,H_Ar_), 6.49 (d, *J* = 8.0 Hz, 1H,H_Ar_), 6.02 (dd, *J* = 9.5, 9.2Hz, 1H,H_Olefin_), 5.86 (d, *J* = 9.9 Hz, 1H,H_Olefin_), 5.22 (d, *J* = 5.7 Hz, 2H,O-CH_2_), 5.19 (s, 1H, O-CH-CH-OH), 4.27 (s, 1H, CH-OH), 2.83 (s, 1H,C_Ar_-CH_2_), 2.81 (s,1H,C_Ar_-CH_2_), 2.53–2.44 (m, 2H,N-CH_2_-), 2.41 (s, 3H, CH_3_), 2.37 (s, 3H,N-CH_3_), 2.33–2.30 (m, 1H,N-CH-), 2.29 (s,1H,C_q_-CH-CH-), 2.07 (s, 1H,N-CH_2_-CH_2_-), 2.04–2.02 (d, *J* = 11.0 Hz, 1H,N-CH_2_-CH_2_-). ^13^C NMR (125 MHz, CDCl_3_) (δ, ppm):146.02, 142.92, 141.22, 139.07, 134.65, 134.53, 132.48, 130.22, 130.20, 129.64, 126.94, 121.51, 120.45, 117.89, 90.65, 66.14, 62.67, 45.28, 45.02, 43.24, 42.91, 37.60, 28.41, 21.10. HRMS (ESI): [M + H]^+^ C_27_H_28_N_4_O_3_ calcd. 457.2239, found 457.5380.

*(4R,4aR,7S,7aR,12bS)-3-methyl-9-((1-phenyl-1H-1,2,3-triazol-4-yl)methoxy)-2,3,4,4a,7,7a-hexahydro-1H-4,12-methanobenzofuro[3,2-e]isoquinolin-7-ol (Compound* ***3j***)

Yield:59%, oil. ^1^H NMR (500 MHz,CDCl_3_) (δ, ppm): 8.07 (s, 1H,H_Triazole_), 7.73 (d, *J* = 8.7 Hz, 2H,H_Ar_),7.71 (d, *J* = 8.9 Hz, 2H,H_Ar_), 7.51 (s, 1H,H_Ar_), 6.68 (d, *J* = 8.0 Hz, 1H,H_Ar_), 6.49 (d, *J* = 8.0 Hz, 1H,H_Ar_), 6.01 (dd, *J* = 9.5,9.3 Hz, 1H,H_Olefin_), 5.86 (d, *J* = 9.9 Hz,1H,H_Olefin_), 5.24 (d, *J* = 6.2 Hz, 2H,O-CH_2_),5.20 (s, 1H, O-CH-CH-OH), 4.26 (s,1H, CH-OH), 2.84 (s, 1H,C_Ar_-CH_2_), 2.70–2.64 (m, 1H,C_Ar_-CH_2_) 2.50–2.33 (m, 2H,N-CH_2_-), 2.29 (s, 3H,N-CH_3_), 2.26–2.23 (m,1H,N-CH-), 2.22–2.19(m,1H,C_q_-CH-CH-), 2.05 (s, 1H,N-CH_2_-CH_2_-),1.99–1.93 (m, 1H,N-CH_2_-CH_2_-). ^13^C NMR (125 MHz, CDCl_3_) (δ, ppm):148.89, 146.67, 140.03, 137.43, 132.53, 130.11, 129.74, 129.69, 129.63, 127.11, 121.47, 120.56, 120.50, 117.80, 90.81, 66.23, 63.70, 62.67, 61.81, 51.86, 45.16, 42.00, 37.42, 32.59. HRMS (ESI): [M + H]^+^ C_26_H_26_N_4_O_3_ calcd. 443.2083, found 443.0531.

*(4R,4aR,7S,7aR,12bS)-9-((1-benzyl-1H-1,2,3-triazol-4-yl)methoxy)-3-methyl-2,3,4,4a,7,7a-hexahydro-1H-4,12-methanobenzofuro[3,2-e]isoquinolin-7-ol (Compound* ***3k***)

Yield:60%, oil. ^1^H NMR (500 MHz,CDCl_3_) (δ, ppm):7.51 (s, 1H,H_Triazole_), 7.35 (d, *J* = 8.2 Hz, 2H,H_Ar_), 7.32 (d, *J* = 8.1 Hz, 2H,H_Ar_), 7.12–7.11 (d, *J* = 7.0 Hz, 1H,H_Ar_), 6.59 (d, *J* = 8.0 Hz, 1H,H_Ar_), 6.44 (d, *J* = 7.9 Hz, 1H,H_Ar_), 5.96 (dd, *J* = 9.5,9.2 Hz, 1H,H_Olefin_), 5.83 (d, *J* = 9.9 Hz,1H,H_Olefin_), 5.50 (d, *J* = 13.7 Hz, 2H,CH_2Benzyl_), 5.14 (d, *J* = 5.5 Hz, 2H,O-CH_2_), 5.09 (s, 1H, O-CH-CH-OH), 4.17 (s, 1H, CH-OH), 2.76 (s, 1H,C_Ar_-CH_2_), 2.60–2.52 (m, 1H,C_Ar_-CH_2_), 2.32–2.24 (m, 2H,N-CH_2_-), 2.20 (s, 3H,N-CH_3_), 2.14–2.11 (m, 1H,N-CH-), 2.10–2.08(m, 1H,C_q_-CH-CH-), 2.01 (s, 1H,N-CH_2_-CH_2_-), 1.91–1.83 (m, 1H,N-CH_2_-CH_2_-). ^13^C NMR (125 MHz, CDCl_3_) (δ, ppm):145.99, 144.37, 140.87, 132.76, 132.62, 129.68, 129.09, 129.08, 128.68, 127.79, 126.85, 125.00, 123.32, 117.59, 90.82, 66.22, 62.76, 54.17, 52.65, 52.12, 45.29, 45.06, 42.08, 37.21, 17.92. HRMS (ESI): [M + H]^+^ C_27_H_28_N_4_O_3_ calcd. 457.2239, found 457.5479.

*(4R,4aR,7S,7aR,12bS)-9-((1-(4-fluorobenzyl)-1H-1,2,3-triazol-4-yl)methoxy)-3-methyl-2,3,4,4a,7,7a-hexahydro-1H-4,12-methanobenzofuro[3,2-e]isoquinolin-7-ol (Compound* ***3l***)

Yield:55%, oil. ^1^H NMR (500 MHz,CDCl_3_) (δ, ppm):7.51 (s, 1H,H_Triazole_), 7.10–7.05 (m, 2H,H_Ar_), 7.00 (m, 2H,H_Ar_), 6.58 (d, *J* = 8.0 Hz, 1H,H_Ar_), 6.44 (d, *J* = 8.0 Hz, 1H,H_Ar_), 5.97 (dd, *J* = 9.5,9.4 Hz, 1H,H_Olefin_), 5.82 (d, *J* = 9.9 Hz, 1H,H_Olefin_), 5.46 (s, 2H, CH_2Benzyl_), 5.14 (d, *J* = 6.3 Hz, 2H,O-CH_2_), 5.09 (s, 1H, O-CH-CH-OH), 4.16 (s, 1H, CH-OH), 2.77 (s, 1H,C_A_r-CH_2_), 2.57–2.48 (m, 1H,C_A_r-CH_2_), 2.30–2.22 (m, 2H,N-CH_2_-), 2.19 (s, 3H,N-CH_3_), 2.12–2.10 (m, 1H,N-CH-), 2.09–2.06 (m, 1H,C_q_-CH-CH-), 2.01–1.93 (m, 1H,N-CH_2_-CH_2_-), 1.91–1.82 (m, 1H,N-CH_2_-CH_2_-).^13^C NMR (125 MHz,CDCl_3_) (δ, ppm): 163.75, 146.73, 144.54, 140.80, 132.78, 129.95, 129.88, 129.66, 129.60, 126.85, 125.00, 123.21, 117.54, 116.18, 90.84, 66.24, 62.75, 53.42, 52.67, 52.20, 45.09, 42.19, 42.18, 37.18, 32.68.HRMS (ESI): [M + H]^+^ C_27_H_27_FN_4_O_3_ calcd. 475.2145, found 475.5500.

*(4R,4aR,7S,7aR,12bS)-9-((1-(4-bromobenzyl)-1H-1,2,3-triazol-4-yl)methoxy)-3-methyl-2,3,4,4a,7,7a-hexahydro-1H-4,12-methanobenzofuro[3,2-e]isoquinolin-7-ol (Compound* ***3m***)

Yield:54%, oil. ^1^H NMR (500 MHz, CDCl_3_) (δ, ppm): 7.36 (s, 1H,H_Triazole_), 7.14 (d, *J* = 8.2 Hz, 2H,H_Ar_), 6.96 (d, *J* = 8.3 Hz, 2H,H_Ar_), 6.58 (d, *J* = 8.0 Hz, 1H,H_Ar_), 6.45 (d, *J* = 8.0 Hz, 1H,H_Ar_), 5.98 (dd, *J* = 9.5, 9.3Hz, 1H,H_Olefin_), 5.81 (d, *J* = 9.9 Hz, 1H,H_Olefin_), 5.44 (m, 2H, CH_2Benzyl_), 5.16–5.11 (m, 2H, O-CH_2_), 5.10 (s, 1H, O-CH-CH-OH), 4.20–4.15 (m, 1H, CH-OH), 2.81 (s, 1H,C_Ar_-CH_2_), 2.76 (m,1H,C_A_r-CH_2_), 2.69–2.64 (m, 2H,N-CH_2_-), 2.62 (s, 3H,N-CH_3_), 2.60–2.56 (m, 1H,N-CH-), 2.57–2.52 (m, 1H,C_q_-CH-CH-), 2.15–2.09 (m, 1H,N-CH_2_-CH_2_-), 1.91–1.85 (m, 1H,N-CH_2_-CH_2_-). ^13^C NMR (125 MHz, CDCl_3_) (δ, ppm): 152.63, 144.78, 143.27, 133.49, 132.27, 132.24, 130.27, 129.65, 129.33, 124.98, 124.96, 123.91, 123.33, 117.56, 104.95, 71.28, 63.70, 62.75, 53.47, 53.41, 47.86, 45.07, 45.04, 38.65, 29.67. HRMS (ESI): [M + H]^+^ C_27_H_27_BrN_4_O_3_ calcd. 535.1345, found 535.1601.

*(4R,4aR,7S,7aR,12bS)-3-methyl-9-((1-(4-methylbenzyl)-1H-1,2,3-triazol-4-yl)methoxy)-2,3,4,4a,7,7a-hexahydro-1H-4,12-methanobenzofuro[3,2-e]isoquinolin-7-ol (Compound* ***3n***)

Yield:53%, oil. ^1^H NMR (500 MHz,CDCl_3_) (δ, ppm):7.32 (s, 1H,H_Triazole_), 7.13 (d, *J* = 7.6 Hz, 2H,H_Ar_), 7.04 (d, *J* = 7.6 Hz, 2H,H_Ar_), 6.59 (d, *J* = 8.0 Hz, 1H,H_Ar_), 5.95 (dd, *J* = 9.5, 9.3 Hz, 1H,H_Olefin_), 5.83 (d, *J* = 9.9 Hz, 1H,H_Olefin_), 5.45 (d, *J* = 11.7 Hz, 2H, CH_2Benzyl_), 5.14–5.13 (d, *J* = 5.7 Hz, 2H,O-CH_2_), 5.12 (d, *J* = 5.3 Hz, 1H, O-CH-CH-OH), 4.17 (s, 1H, CH-OH), 2.76 (s, 1H,C_Ar_-CH_2_), 2.61–2.54 (m, 1H,C_Ar_-CH_2_), 2.45–2.38 (m, 2H,N-CH_2_-), 2.34 (s, 3H,CH_3_), 2.30–2.28 (m, 1H,N-CH-),2.27–2.24 (m, 1H,C_q_-CH-CH-), 2.20 (s, 3H,N-CH_3_), 2.12–2.08 (m, 1H,N-CH_2_-CH_2_-), 1.92–1.85 (m, 1H,N-CH_2_-CH_2_-). ^13^C NMR (125MHz, CDCl_3_) (δ, ppm): 147.49, 140.93, 139.31, 138.59, 132.75, 129.74, 129.66, 128.10, 127.93, 126.84, 124.99, 123.18, 117.59, 90.77, 66.21, 63.19, 62.72, 54.02, 53.91, 52.51, 45.04, 41.94, 37.19, 21.14, 21.12. HRMS (ESI): [M + H]^+^ C_28_H_30_N_4_O_3_ calcd. 471.2396, found 471.3356.

### HPLC analysis

The chromatographic separation was performed by using a mobile phase B: acetonitrile and mobile phase A: ammonium acetate buffer (60 mM, pH = 3). For sepration of the synthesized compounds a gradient method was used. The eluent started at 100% acetonitrile, gradually decreasing to 95% acetonitrile over 10 minute, then from minute 10–15 the eluent was decreased to 85% acetonitrile. Finally the method was run isocratically from minute 15 to minute 40 with 85% acetonitrile. The flow rate was maintained at 1.5 mL/min and detection was performed at wavelength 254 nm. The data was processed using empower2. The 1 mg of **3d** was dissolved in 1 ml 90%ACN:10% buffer acetate and The 1 mg of **3b** was dissolved in 1 ml 90% ACN:10% buffer and then injected into HPLC.

### Biological assay

To obtain more specific results on the efficacy of the compounds, the animal behavioral test was conducted. Among different tests of acute pain assessments, the tail-flick test was chosen which was defined by D’amour and Smith in 1941 [52].

### Animals

NMRI male mice with an approximate weight of 20–25 g were expended in this test. Animals were used only once and were housed in groups of 8 and had free access to food and water. They were kept in a 12-hour light-dark cycle, at a temperature of 22 ± 2 centigrade degree, and a humidity of 45–50%. In the case of compatibility with the environment, all the animals were handled in the lab every day for a week before the experiment and one hour before starting the tail-flick exam. We did our best to minimize the number of animals and reduce the discomfort of them. In the following, the animal experimentation ethics committee of Shahid Beheshti University of Medical Sciences approved that this study was carried out following the NIH Animal Care and Use Committee Guide for the Care and Use of Laboratory Animals and standards for conducting medical research in Iran (Approval ID: IR.SBMU.RETECH.REC.1403.653). All animals were euthanized using CO_2_ inhalation, following the American Veterinary Medical Association (AVMA) guidelines for euthanasia. The chamber was gradually filled with CO_2_ at a rate of 30% per minute until the animals fell unconscious.

### Sample’s preparation

For each novel compound and morphine (positive control), 4 doses of 4, 2, 1, and 0.5 mg/kg were used. These concentrations were selected based on previous studies [53,54]. Also, for the positive control group, 4 doses of morphine were used.

### Tail-flick test

Each mouse was held in a restrainer in a way that the tail was out of the restrainer and additional movement of the animal was reduced. A thermal light was radiated to the middle third of the tail. The pain produced by the light led to a motor reflex in which animals move their tails and take out from the light beam. In the following, the light was shut down, and the chronometer calculated the time needed to create a thermal reflex. The average of three consecutive measurements was registered as “tail flick latency”. In more detail, this factor was used as the thermal pain index, which helps in pain measurement. To avoid serious injuries in animals, the cut-off time was set at 10 seconds. For the evaluation of the compound effect, the tail-flick test was carried out in two steps. Each mouse was compared with itself before medicine consumption. In that case, in the first step tail-flick test was conducted before medicine reception and was registered as “control reaction time (CRT)”. In the following, the second measurement of tail latency was held 30 minutes after compound injections and was called “test reaction time (TRT)”. Each solution was injected into groups of 8 mice via the subcutaneous route [55].

### Naloxone test

To prove the interaction of the novel compounds with opioid receptors, naloxone hydrochloride was used as an opioid antagonist. After the control reaction time assessment, 1mg/kg of naloxone was injected through the intraperitoneal way [41]. After 10 minutes, the studied compound with a dose of 4 mg/kg was injected subcutaneously [42]. The test reaction time was evaluated 30 minutes after the injection of the novel compounds.

### ADME/T studies

ADME/T properties are an essential factor for determining the pseudo-pharmaceutical properties of compounds [56]. This highlights the significance of these studies in drug discovery. Therefore, in this research, the pharmacological similarity and pharmacokinetic parameters of the compounds were predicted using the SwissADME web server [57].

### Statistical analyses

The statistical analyses were done with GraphPad Prism (ver 9.4.1); all of the data were reported in mean±SEM. The data in each group passed the D’Agostino & Pearson normality test and for group comparisons, One-way ANOVA with Tukey’s post hoc test were applied. Tail flick results were calculated as “MPE index” which expresses the maximum possible effect of studied compounds as a percentage and calculated via [(TRT - CRT)/(Cut-off time – CRT)]×100 [58]. Also, for potency comparison, ED_50_ was calculated by non-linear regression analysis and was informed in mean mg/kg with a 95% confidence interval.

## Limitation

As the first step, this study is dedicated to the synthesis and efficacy screening of the novel 1,2,3-triazole tethered derivatives of morphine compounds. It is a preliminary exploration of these novel compounds, with the understanding that the aim of this study was not to investigate toxicity or clinical studies. The tail-flick test was utilized to evaluate acute pain relief. However, additional models, such as the formalin test, contributed to understanding acute and inflammatory pain. The formalin test was excluded from this study due to ethical considerations and a specific focus on inflammatory pain. This study primarily assessed the potency of compounds by measuring their ED_50_ values. Future research will aim to explore persistent and inflammatory pain relief, assess acute and chronic toxicity, and conduct pharmacokinetic evaluations to ensure safety and clarify the compounds’ therapeutic index. Moreover, the future of drug discovery is optimistic, with a roadmap that includes (a) *in vitro* studies such as cytotoxicity screening, mutagenicity assessments, organ-specific toxicity evaluations, reactive oxygen species (ROS) studies, and immune response profiling and (b) *in vivo* studies, including acute/subacute/chronic toxicity, long-lasting and inflammation effects of the compounds, and pharmacodynamic (PD) studies, and (c) optimization of drug delivery by carrying out preclinical formulation tests, and Regulatory and Translational Studies, are all principal factors of these researchers.

## Supporting information

S1 Fig Spectral data of compound 2. (PDF)

S2 Fig Spectral data of compound 3a. (PDF)

S3 Fig Spectral data of compound 3b. (PDF)

S4 Fig Spectral data of compound 3c. (PDF)

S5 Fig Spectral data of compound 3d. (PDF)

S6 Fig Spectral data of compound 3e. (PDF)

S7 Fig Spectral data of compound 3f. (PDF)

S8 Fig Spectral data of compound 3g. (PDF)

S9 Fig Spectral data of compound 3h. (PDF)

S10 Fig Spectral data of compound 3i. (PDF)

S11 Fig Spectral data of compound 3j. (PDF)

S12 Fig Spectral data of compound 3k. (PDF)

S13 Fig Spectral data of compound 3l. (PDF)

S14 Fig Spectral data of compound 3m. (PDF)

S15 Fig Spectral data of compound 3n. (PDF)

S16 Fig Graphs of anti-nociceptive effects of all compounds in the Tail-flick test. (PDF)

S17 Table Raw data of all compounds in the tail-flick test. (PDF)
