# Supplementary figures and images for: Design, synthesis, in-vivo, and in-silico studies of 1,2,3-triazole tethered derivatives of morphine as novel anti-nociceptive agents
Source: PLoS One. 2025 Jun 16;20(6):e0323189. doi: 10.1371/journal.pone.0323189 (PMC12169543; doi:10.1371/journal.pone.0323189)

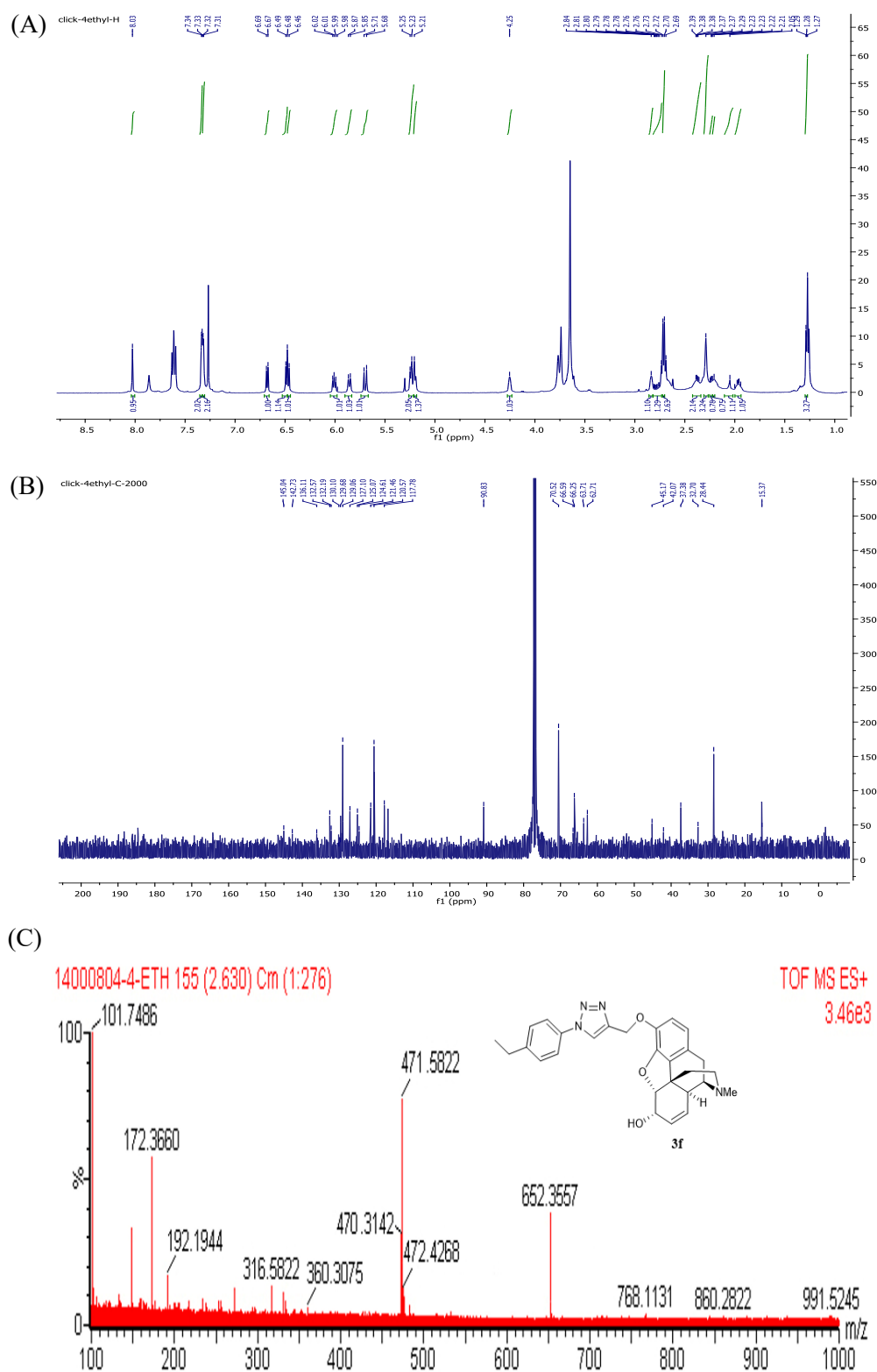

Supplement: S7 Fig — (PDF) [file pone.0323189.s007.pdf]
