# Supplementary material for: Design, synthesis, in-vivo, and in-silico studies of 1,2,3-triazole tethered derivatives of morphine as novel anti-nociceptive agents
Source: PLoS One. 2025 Jun 16;20(6):e0323189. doi: 10.1371/journal.pone.0323189 (PMC12169543; doi:10.1371/journal.pone.0323189)
Supplement: S12 Fig — (PDF) [file pone.0323189.s012.pdf]

(A)

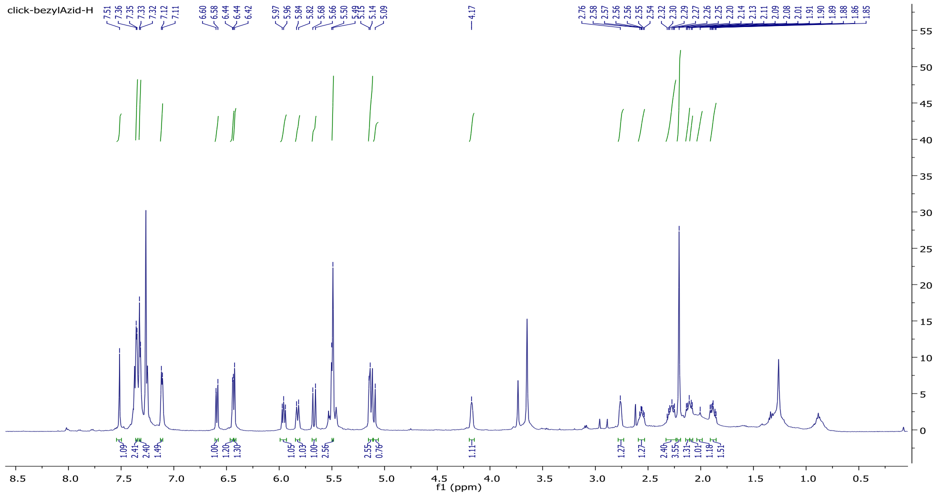

(B)

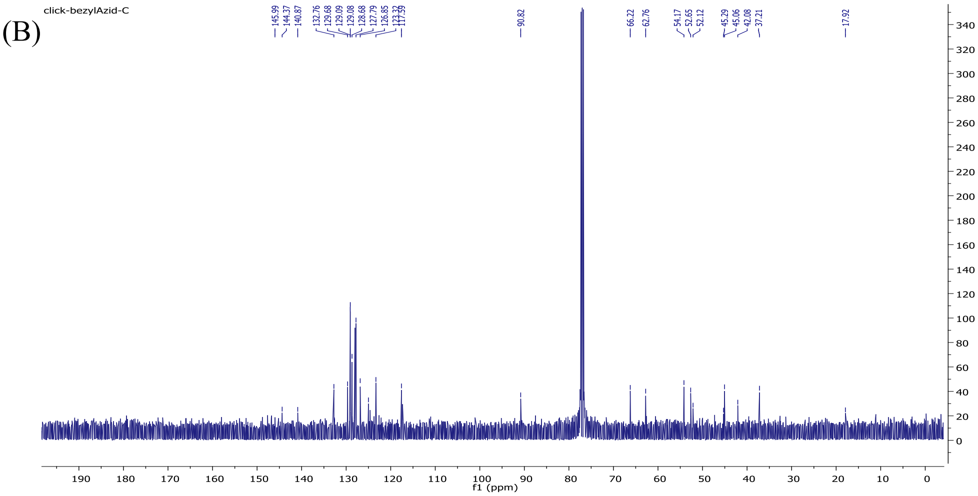

(C)

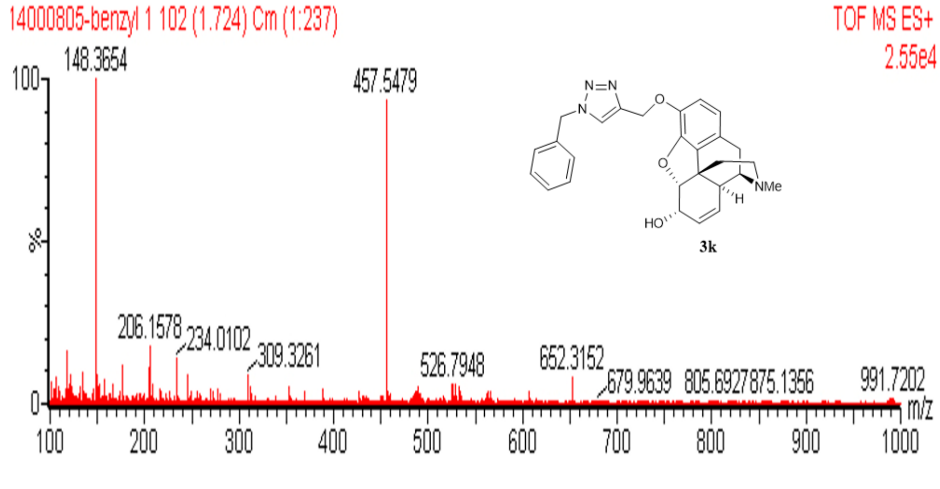

S12 Fig. Spectral data of compound 3k. (A) ^{1}HNMR spectrum of compound 3k. (B) ^{13}CNMR spectrum of compound 3k. (C) Mass spectrum of compound 3k.
