# Supplementary material for: Design, synthesis, in-vivo, and in-silico studies of 1,2,3-triazole tethered derivatives of morphine as novel anti-nociceptive agents
Source: PLoS One. 2025 Jun 16;20(6):e0323189. doi: 10.1371/journal.pone.0323189 (PMC12169543; doi:10.1371/journal.pone.0323189)
Supplement: S16 Fig — (PDF) [file pone.0323189.s016.pdf]

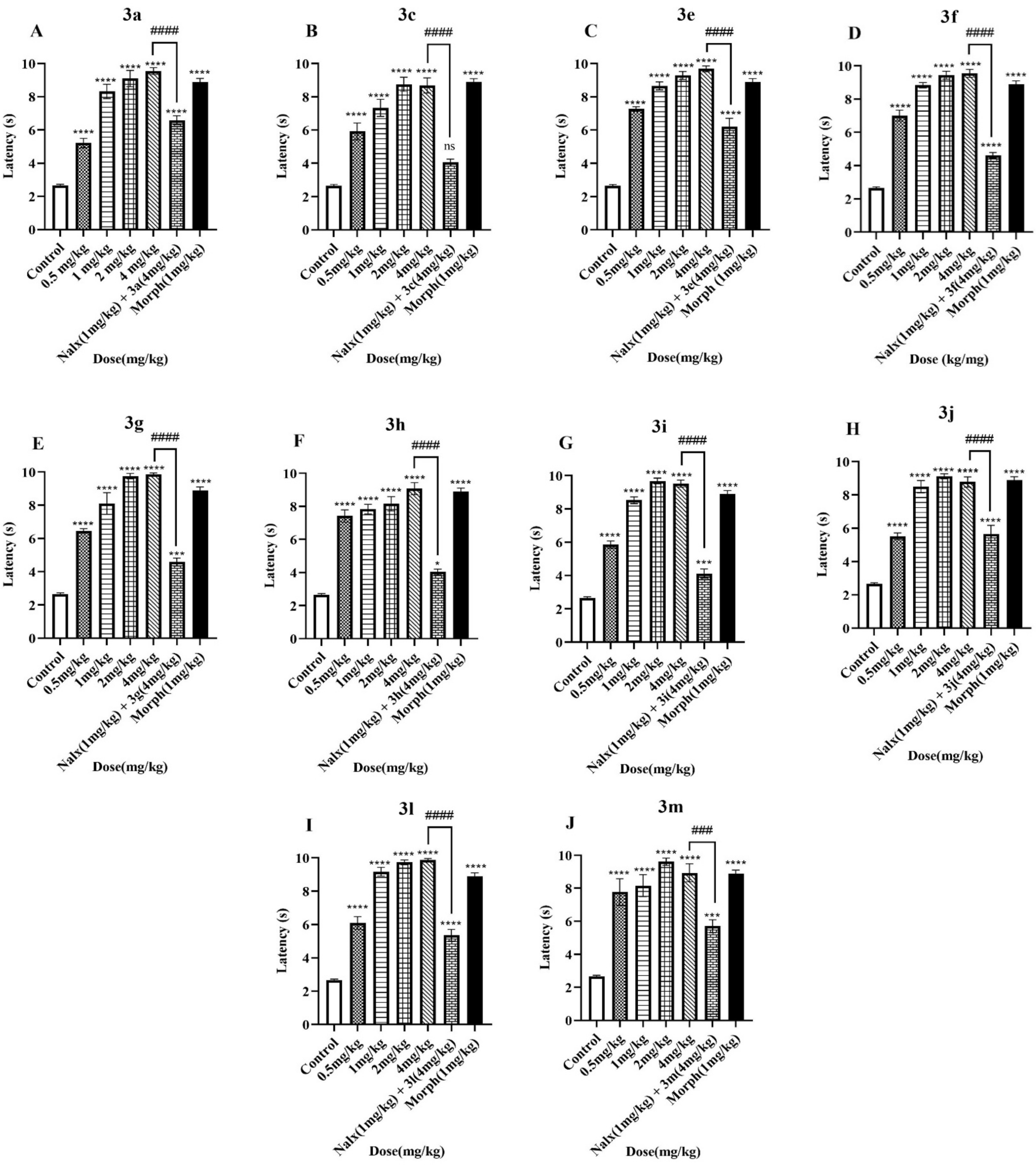

S16 Fig. Graphs of anti-nociceptive effects of all compounds in the tail-flick test. Comparison of the anti-nociceptive effects of four triazole derivatives of morphine with negative and positive control groups in the tail-flick test is demonstrated in (A) 3a, (B) 3c, (C) 3e, (D) 3f, (E) 3g, (F) 3h, (G) 3i, (H) 3j, (I) 3l, and (J) 3m. Results are indicated as Mean ± SEM of 8 separate animals in each group. ****p≤0.0001, ####p≤0.0001.
