# Supplementary material for: Design, synthesis, in-vivo, and in-silico studies of 1,2,3-triazole tethered derivatives of morphine as novel anti-nociceptive agents
Source: PLoS One. 2025 Jun 16;20(6):e0323189. doi: 10.1371/journal.pone.0323189 (PMC12169543; doi:10.1371/journal.pone.0323189)
Supplement: S17 Table — (PDF) [file pone.0323189.s017.pdf]

S17 Table. **Raw data of all compounds in the tail-flick test.** Raw data for tail-flick latency test in mice treated with different doses (0.5, 1, 2 and 4 mg/kg) of all compounds. The table shows control response times (CRT), treatment response times (TRT), and percentage of maximal possible effect (%MPE) for each dose group. Data were collected from eight individual animals per group.

Compounds	No. of animals	0.5 mg/kg			1 mg/kg			2 mg/kg			4 mg/kg			4 mg/kg + Naloxone		
		CRT	TRT	%MPE	CRT	TRT	%MPE	CRT	TRT	%MPE	CRT	TRT	%MPE	CRT	TRT	%MPE
Compound **3a**	1	2.4	4.5	28.7	2.2	6.4	53.8	2.0	10	100	2.4	10	100	2.4	7.0	60.5
	2	2.6	4.8	30.2	2.3	8.5	80.5	2.2	10	100	1.9	8.6	82.7	2	6.5	56.2
	3	2.0	4.5	31.2	1.9	8.1	77.7	2.5	10	100	1.9	9.3	91.3	2.4	7.2	63.1
	4	1.9	5.4	43.2	2.0	9.7	96.2	1.9	9.7	97.4	2.7	10	100	2.5	6.6	54.6
	5	2.6	6.1	47.3	2.5	7.7	70.3	2.3	9.2	89.6	2.0	10	77.0	2.5	6.9	58.6
	6	2.3	5.7	44.1	1.9	8.9	86.4	2.2	7.3	65.8	1.9	9.7	96.3	2.3	5.0	35.0
	7	2.0	6.1	51.3	2.2	10	100	1.9	6.9	62.9	2.2	10	100	2.4	7.4	65.7
	8	2.6	4.6	27.0	1.9	7.1	65.3	2.1	9.4	93.5	2.3	8.8	100	2.4	6.1	48.6
Compound **3b**	1	2.6	6.9	58.1	2.4	9.8	98.6	2.6	10	100	2.3	10	100	2.7	3.7	13.7
	2	2.2	7.2	64.1	2.3	9.8	97.6	2.7	10	100	2.2	10	100	2.1	3.8	21.5
	3	2.0	5.1	38.7	1.8	8.4	81.2	1.9	10	100	2.3	10	100	1.9	3.4	18.5
	4	2.6	5.8	43.2	2.0	7.5	68.9	2.6	10	100	2.4	8.9	85.5	2.6	4.8	29.7
	5	2.0	7.5	69.2	2.8	7.7	68.7	2.3	10	100	2.4	10	100	2.0	3.6	20.0
	6	2.0	8.4	80.0	2.4	7.5	67.5	2.7	9.6	95.8	2.3	9.9	98.7	2.4	3.4	13.1
	7	2.4	6.9	59.2	1.9	10	100	2.7	9.6	94.5	2.4	9.3	90.7	2.0	3.1	13.7
	8	2.2	7.6	69.2	2.2	10	100	2.3	9.5	93.5	2.3	9.7	96.1	1.9	4.1	27.1
Compound **3c**	1	1.6	7.0	65.4	2.5	8.1	74.6	2.3	8.7	83.1	2.0	10	100	2.0	4.3	28.7
	2	2.0	4.1	26.2	2.2	7.2	64.1	2.3	8.9	85.7	2.0	10	100	2.3	4.2	25.9
	3	1.9	5.4	43.2	2.3	6.6	55.8	2.7	9.9	98.6	2.4	9.4	92.1	2.7	4.7	27.4
	4	2.3	6.6	55.8	2.0	8.7	83.7	2.0	10	100	1.8	9.5	93.9	2.2	4.9	34.6
	5	1.9	5.1	39.5	2.4	9.6	94.7	2.4	7.2	63.1	2.3	8.8	85.0	2.4	3.4	13.1
	6	2.0	6.7	58.7	2.4	4.6	29.3	2.2	7.0	61.5	2.2	7.0	61.5	1.8	3.9	25.6
	7	2.6	8.1	75.6	2.4	5.4	40.7	2.0	8.4	80.0	2.4	7.8	72.3	2.4	3.7	17.1
	8	2.3	4.1	24.6	2.3	7.5	67.5	2.2	10	100	2.3	8.9	85.7	2.4	3.2	10.5

S17 Table. Continued from pervious page.

Compounds	No. of animals	0.5 mg/kg			1 mg/kg			2 mg/kg			4 mg/kg			4 mg/kg + Naloxone		
		CRT	TRT	%MPE	CRT	TRT	%MPE	CRT	TRT	%MPE	CRT	TRT	%MPE	CRT	TRT	%MPE
Compound **3d**	1	1.2	6.8	63.6	2.4	8.5	81.5	2.0	9.9	98.7	1.5	9.2	91.7	2.5	5.0	33.3
	2	1.2	7.0	65.9	2.4	8.6	82.8	2.2	8.9	87.1	1.9	10	100	2.4	4.8	32.8
	3	1.6	7.2	67.5	2.0	9.1	88.7	2.0	10	100	1.9	10	100	2.2	5.3	39.7
	4	2.3	6.0	48.0	2.0	9.0	87.5	2.2	10	100	2.0	10	100	2.5	5.7	42.6
	5	1.4	6.5	59.3	2.4	8.5	81.5	1.9	8.6	82.7	2.2	10	100	1.6	5.5	46.4
	6	2.2	6.6	57.6	2.4	8.6	82.8	2.0	10	100	2.0	9.5	93.7	2.2	5.4	41.0
	7	2.6	6.7	55.4	2.0	9.1	88.7	1.8	10	100	2.0	10	100	2.0	6.2	52.5
	8	2.4	7.8	72.3	2.0	9.0	87.5	2.2	10	100	4.2	10	100	2.8	5.1	31.9
Compound **3e**	1	1.9	7.2	66.6	2.0	9.2	90.0	2.0	8.1	76.2	2.4	9.7	97.3	1.6	5.8	50.0
	2	1.5	7.2	67.0	2.4	8.3	77.6	1.9	9.7	96.3	2.0	9.2	90.0	2.2	7.7	70.5
	3	1.6	7.8	73.8	2.0	9.1	88.7	2.8	8.3	77.7	1.9	8.7	83.9	2.4	7.3	65.7
	4	2.2	7.7	70.5	2.2	8.3	79.4	2.6	9.3	91.8	2.0	10	100	1.9	8.0	75.3
	5	2.4	6.7	57.8	2.3	9.2	89.6	2.0	10	100	2.2	10	100	2.2	5.8	46.1
	6	2.2	6.7	58.9	2.3	9.3	90.9	2.3	9.7	96.1	1.9	10	100	1.6	6.1	54.7
	7	2.0	7.6	70.0	2.3	8.5	80.5	2.3	9.7	96.1	2.3	10	100	1.8	4.9	37.8
	8	2.3	7.0	61.0	2.4	7.2	64.4	1.9	9.3	91.3	2.0	10	100	1.8	3.7	24.3
Compound **3f**	1	2.3	8.1	75.3	2.7	8.8	83.5	2.3	10	100	2.3	10	100	2.3	5.0	35.0
	2	2.7	8.0	72.6	2.0	8.7	83.7	2.2	8.8	84.6	2.2	9.2	89.7	2.0	5.4	42.5
	3	2.4	5.7	43.4	2.0	8.8	85.0	2.2	10	100	2.3	8.4	79.9	2.0	4.5	31.2
	4	2.7	6.5	52.0	2.4	8.1	75.0	2.6	10	100	2.0	10	100	1.9	4.6	33.3
	5	2.3	6.9	59.7	2.2	8.7	83.3	2.6	8.7	82.4	2.6	10	100	2.3	5.0	35.0
	6	2.2	7.0	61.5	2.2	8.8	84.6	2.4	9.7	96.0	2.1	8.8	86.0	2.4	4.3	25.0
	7	2.2	6.0	48.7	1.9	9.0	88.8	2.5	9.9	98.6	2.4	10	100	2.0	4.2	27.5
	8	1.5	7.8	75.2	2.0	9.7	96.2	2.5	8.4	78.6	2.6	10	100	2.7	4.0	17.8
Compound **3g**	1	1.8	6.2	54.8	2.2	10	100	2.0	10	100	2.0	9.4	92.5	2.3	5.5	41.5
	2	2.3	5.8	45.4	2.6	10	100	2.0	10	100	1.4	9.6	95.3	2.0	4.3	28.7
	3	2.0	6.2	52.5	2.0	10	100	1.6	9.0	89.2	2.2	10	100	1.7	5.0	40.9
	4	2.0	6.3	54.1	2.1	9.1	89.8	2.2	8.9	87.1	1.8	10	100	2.2	4.7	32.0
	5	2.2	6.4	53.8	2.1	6.7	59.4	1.5	10	100	1.5	10	100	2.7	4.0	17.8
	6	2.8	7.3	62.5	2.0	6.4	55	2.3	10	100	1.9	10	100	2.0	3.5	18.7
	7	2.0	6.8	60	1.8	5.9	50	1.6	10	100	2.0	9.9	98.7	2.0	4.3	28.7
	8	2.0	6.5	56.2	2.2	6.6	56.4	2.2	10	100	1.5	10	100	2.0	5.3	41.2

S17 Table. Continued from pervious page.

Compounds	No. of animals	0.5 mg/kg			1 mg/kg			2 mg/kg			4 mg/kg			4 mg/kg + Naloxone		
		CRT	TRT	%MPE	CRT	TRT	%MPE	CRT	TRT	%MPE	CRT	TRT	%MPE	CRT	TRT	%MPE
Compound **3h**	1	2.2	4.7	32.0	2.6	7.5	66.2	1.7	7.1	66.2	2.3	10	100	2.4	4.3	25.0
	2	2.4	6.2	50.0	2.0	6.9	61.2	2.0	8.3	78.7	1.9	8.3	79.0	1.9	4.6	34.5
	3	2.3	6.8	58.4	2.2	7.2	65.3	2.7	9.6	94.5	2.0	10	100	2.3	4.5	29.8
	4	2.6	7.4	64.8	2.8	9.0	86.1	1.8	9.3	92.6	2.3	10	100	2.3	4.1	24.6
	5	2.0	6.5	56.2	2.2	8.0	74.3	2.3	6.0	48.0	2.4	8.6	82.8	2.3	3.7	19.4
	6	2.0	6.1	51.2	2.4	8.3	77.6	2.0	8.9	86.5	2.8	10	100	2.4	3.7	17.1
	7	1.9	4.9	37.0	1.9	6.9	61.7	1.9	7.4	67.9	1.9	7.8	74.0	2.2	3.2	12.8
	8	2.6	4.6	27.0	2.3	8.8	84.4	1.9	8.4	81.4	2.2	7.7	70.5	2.0	3.8	22.5
Compound **3i**	1	1.9	6.4	55.5	2.7	8.0	72.6	2.7	10	100	2.0	10	100	2.0	4.1	26.2
	2	2.2	5.0	37.1	2.0	8.5	81.2	2.3	10	100	2.3	9.4	92.2	2.3	4.1	24.6
	3	2.7	6.8	56.1	2.0	9.7	96.2	2.3	10	100	2.2	10	100	2.5	4.5	26.6
	4	2.2	5.4	41.0	2.8	8.0	72.2	1.9	9.0	88.8	2.8	8.9	84.7	2.2	5.5	43.5
	5	2.0	5.8	47.5	2.2	8.9	85.9	2.6	9.8	97.3	2.3	10	100	2.2	3.4	16.6
	6	2.4	6.5	53.9	2.2	8.0	74.3	2.8	8.7	81.9	2.3	8.4	79.2	2.3	3.7	19.4
	7	2.6	5.4	39.1	2.0	8.7	83.7	2.4	9.9	98.6	2.2	9.4	93.5	2.8	2.9	1.39
	8	2.8	5.4	36.1	2.6	8.3	78.3	2.3	9.8	98.7	2.3	10	100	1.9	4.3	30.8
Compound **3j**	1	2.0	6.1	51.2	2.4	7.2	64.4	2.0	8.8	85.0	2.2	9.1	88.4	2.4	5.0	34.2
	2	2.2	5.3	39.7	2.4	7.3	65.7	2.3	8.9	85.7	2.5	9.1	88.0	2.0	5.8	47.5
	3	2.6	5.4	37.8	2.5	8.8	84.0	1.8	9.6	95.1	1.9	8.7	83.9	2.2	6.4	53.8
	4	2.2	6.1	50.0	2.2	9.3	91.0	2.3	9.9	98.7	2.4	9.1	89.4	2.9	6.8	54.9
	5	2.2	4.4	29.4	2.3	10	100	2.2	8.7	83.3	2.2	10	100	2.3	3.1	11.6
	6	2.0	5.0	37.5	2.6	8.7	82.4	2.3	9.1	88.3	1.9	6.9	62.9	2.8	4.0	16.6
	7	2.2	6.1	50.0	2.4	7.2	64.4	2.3	8.8	84.4	2.3	8.8	84.4	2.4	7.2	64.4
	8	2.3	5.7	44.1	2.4	9.1	89.4	2.2	9.2	89.7	2.7	8.3	78.0	2.6	7.0	59.4
Compound **3k**	1	2.8	7.0	58.3	2.7	10	100	2.4	10	100	2.2	10	100	2.7	4.7	27.4
	2	2.3	8.4	79.2	2.6	10	100	2.2	10	100	2.0	10	100	2.7	5.7	42.2
	3	1.9	8.1	77.7	2.2	10	100	2.7	10	100	2.6	10	100	2.6	4.9	31.0
	4	2.4	7.2	63.1	2.2	10	100	2.7	10	100	2.6	9.1	87.8	2.4	5.1	35.5
	5	2.0	7.3	66.2	2.3	7.9	72.7	2.7	8.5	79.4	2.0	9.7	96.2	2.8	5.6	38.8
	6	2.3	6.2	50.6	2.5	10	100	2.4	6.6	55.2	2.7	9.3	91.7	2.5	4.8	30.6
	7	2.3	8.7	83.1	2.6	5.4	39.1	2.3	9.2	89.6	2.8	10	100	2.8	3.9	15.2
	8	1.9	7.2	65.4	2.4	5.3	39.4	2.3	8.0	74.0	2.3	9.2	89.6	2.2	5.0	35.9

S17 Table. Continued from pervious page.

Compounds	No. of animals	0.5 mg/kg			1 mg/kg			2 mg/kg			4 mg/kg			4 mg/kg + Naloxone		
		CRT	TRT	%MPE	CRT	TRT	%MPE	CRT	TRT	%MPE	CRT	TRT	%MPE	CRT	TRT	%MPE
Compound **3l**	1	2.3	5.8	45.4	2.7	9.1	87.6	2.4	10	100	1.8	10	100	2.0	5.4	42.5
	2	2.6	5.4	37.8	2.0	9.7	96.2	2.6	10	100	2.4	10	100	2.2	5.4	41.0
	3	2.2	5.2	38.4	1.9	10	100	2.4	8.9	85.5	2.4	10	100	2.4	5.7	43.4
	4	1.9	7.4	67.9	2.3	10	100	2.0	10	100	2.4	9.4	92.1	2.3	6.4	53.2
	5	1.9	4.9	37.0	2.3	8.1	75.3	1.9	9.5	93.8	2.7	9.6	94.5	2.3	6.2	50.6
	6	2.4	7.0	60.5	2.2	8.4	80.7	2.3	10	100	2.6	10	100	2.3	6.1	49.3
	7	2.0	5.5	43.7	2.6	9.5	93.2	2.6	9.5	93.2	2.4	10	100	2.4	3.9	19.7
	8	2.2	7.6	69.2	2.2	8.4	80.7	1.9	10	100	2.4	10	100	2.6	3.8	16.2
Compound **3m**	1	2.4	8.6	82.8	2.6	7.6	67.5	2.4	10	100	2.4	10	100	2.7	5.2	34.2
	2	2.4	10	100	2.3	10	100	2.7	10	100	2.3	10	100	2.4	7.2	63.1
	3	2.7	10	100	2.8	9.1	88.8	2.3	10	100	2.9	9.8	98.5	2.7	7.2	61.8
	4	3.0	8.1	72.8	2.8	9.4	91.8	2.8	10	100	2.0	10	100	2.7	6.8	56.1
	5	2.0	4.9	36.2	2.0	10	100	2.2	10	100	2.8	10	100	2.3	7.7	70.1
	6	3.0	9.9	98.5	2.8	6.7	54.1	1.9	9.0	87.6	2.7	6.9	58.0	1.9	6.1	53.0
	7	2.3	5.3	38.9	2.0	7.5	68.7	2.6	8.3	78.3	2.6	8.3	78.0	2.6	3.8	16.2
	8	2.5	5.3	37.3	2.2	4.7	32.0	2.2	9.4	93.5	1.9	6.1	53.0	2.7	4.5	24.6
Compound **3n**	1	2.2	6.6	57.6	2.4	6.6	56.5	1.9	9.2	90.1	2.7	10	100	2.6	7.7	68.9
	2	2.4	3.9	19.7	1.5	6.1	54.1	2.2	8.4	80.7	2.8	8.2	75.0	2.6	8.7	82.4
	3	2.3	4.5	29.8	2.3	7.5	67.5	2.1	9.1	88.6	1.8	10	100	1.9	6.0	50.6
	4	1.9	4.1	27.1	2.2	7.7	70.5	2.2	9.5	94.8	2.0	8.5	81.2	3.0	6.0	42.8
	5	2.0	4.6	32.5	2.4	7.9	73.6	1.9	9.2	90.1	2.3	9.9	98.7	2.2	6.6	57.6
	6	2.5	6.9	58.6	2.3	5.4	40.2	2.4	9.1	88.1	2.7	10	100	2.6	4.2	21.6
	7	2.6	6.8	56.7	2.3	7.6	68.8	2.5	8.5	80.0	2.7	9.6	95.8	2.5	7.0	60.0
	8	1.5	5.5	47.0	2.7	4.9	30.1	2.5	7.4	65.3	2.4	10	100	2.7	7.8	69.8
